# Mutations in Barley Row Type Genes Have Pleiotropic Effects on Shoot Branching

**DOI:** 10.1371/journal.pone.0140246

**Published:** 2015-10-14

**Authors:** Corinna Brit Liller, René Neuhaus, Maria von Korff, Maarten Koornneef, Wilma van Esse

**Affiliations:** 1 Department Plant Breeding and Genetics, Max Planck Institute for Plant Breeding Research, Carl-von-Linné-Weg 10, 50829, Köln, Germany; 2 Institute for Plant Genetics, Heinrich-Heine-Universität Düsseldorf, Universitätsstr. 1, 40225, Düsseldorf, Germany; 3 Cluster of Excellence in Plant Sciences (CEPLAS), Heinrich-Heine-Universität Düsseldorf, Universitätsstr. 1, 40255, Düsseldorf, Germany; 4 Laboratory of Genetics, Wageningen University, Droevendaalsesteeg 1, 6708 PB Wageningen, the Netherlands; University of Manitoba, CANADA

## Abstract

Cereal crop yield is determined by different yield components such as seed weight, seed number per spike and the tiller number and spikes. Negative correlations between these traits are often attributed to resource limitation. However, recent evidence suggests that the same genes or regulatory modules can regulate both inflorescence branching and tillering. It is therefore important to explore the role of genetic correlations between different yield components in small grain cereals. In this work, we studied pleiotropic effects of row type genes on seed size, seed number per spike, thousand grain weight, and tillering in barley to better understand the genetic correlations between individual yield components. Allelic mutants of nine different row type loci (36 mutants), in the original spring barley varieties Barke, Bonus and Foma and introgressed in the spring barley cultivar Bowman, were phenotyped under greenhouse and outdoor conditions. We identified two main mutant groups characterized by their relationships between seed and tillering parameters. The first group comprises all mutants with an increased number of seeds and significant change in tiller number at early development (group 1a) or reduced tillering only at full maturity (group 1b). Mutants in the second group are characterized by a reduction in seeds per spike and tiller number, thus exhibiting positive correlations between seed and tiller number. Reduced tillering at full maturity (group 1b) is likely due to resource limitations. In contrast, altered tillering at early development (groups 1a and 2) suggests that the same genes or regulatory modules affect inflorescence and shoot branching. Understanding the genetic bases of the trade-offs between these traits is important for the genetic manipulation of individual yield components.

## Introduction

**Fig 1:**
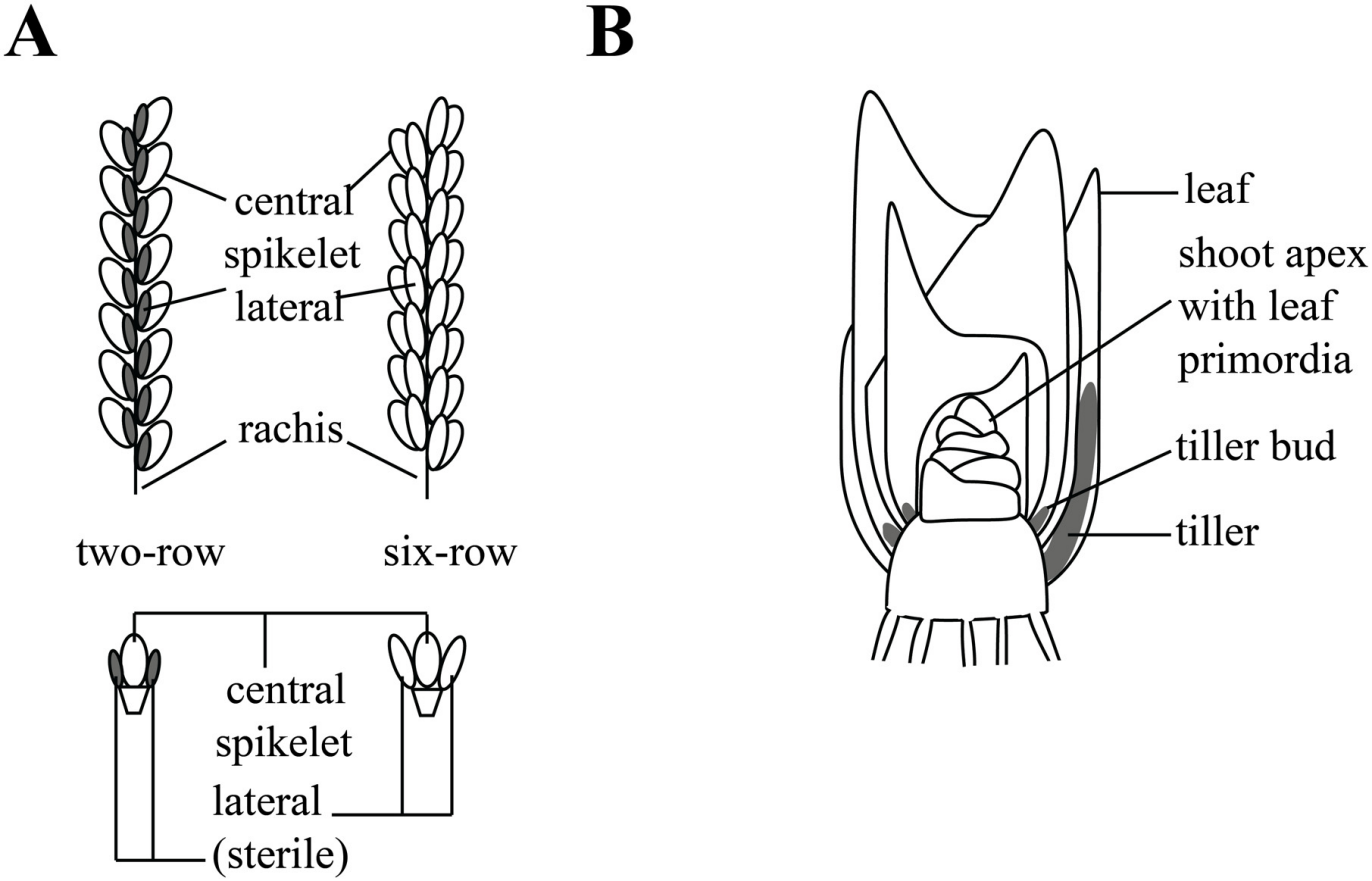
Schematic representation of barley inflorescence architecture. (**A**) Branched grass inflorescence architectures of; two-rowed and six-rowed barley (*H*. *vulgare*) spike. (**A** bottom) Single spike nodes of two- and six-rowed spikes. (**B**) Representation of the shoot architecture found in grass species.

Barley (*Hordeum vulgare*) is one of the most important cereal crops used for food and feed production [1]. In cereals, grain yield can be broken down to three main components: (1) spike number per plant, (2) grain number per spike, and (3) Thousand Grain Weight (TGW) [2,3]. The barley inflorescence (spike) can appear in two main shapes: two-rowed and six-rowed (Fig 1A). In two-rowed genotypes only the central spikelet develops and the two laterals remain sterile or staminate. In six-rowed cultivars, all three spikelets are fully fertile and develop into grains, leading to an increase in grain number per spike [4]. Over the last decades different loci that affect row type have been identified in barley. The loci have been termed *six-rowed spike* (*vrs*), *hexastichon* (*hex-v*) or *intermedium spike* (*int*), depending on the mutant screen and the phenotype [5,6]. *Intermedium spike* mutants typically have a phenotype in between a two-rowed and a fully developed six-rowed spike [5–7]. The spike phenotype of six-rowed barley cultivars mainly relies on mutations in the *Vrs1* (allelic with *Hex-v* and *Int-d*) and *Vrs4* loci. The gene underlying the *Vrs1* locus encodes a HD-ZIP transcription factor (*HvHox1*) which is expressed in the lateral spikelet primordia in the developing spike. *Vrs1* suppresses lateral spikelet fertility resulting in the two-rowed phenotype [8]. Similar to *vrs1* mutants, the *vrs4* mutant shows complete fertility of central and lateral spikelets and the spikes produce additional florets. *Vrs4* encodes a homolog of maize *RAMOSA 2* (*RA2*) a gene involved in the control of branching in the female inflorescence (ear) [9,10]. An increase in seed number often results in a decrease in TGW [3,11]. In addition, the six-rowed spike phenotype has been described to negatively affect tiller number under field conditions [3,12]. Negative correlations between seed number, TGW and tillering have hampered the genetic improvement of overall yield. The opposing effects between these yield components are often attributed to limitations in amounts of assimilates available [3]. Tiller number is mainly affected by the initiation and outgrowth of axillary meristems (AM) and depends on endogenous developmental, hormonal and environmental signals [13–16]. The plant shoot apical meristem (SAM) coordinates the formation of all above ground organs such as leaves, internodes, inflorescence and AMs (Fig 1). During vegetative development, the AM initiates several leaf primordia, which form an axillary bud. In grasses, the axillary meristems capable of giving rise to side shoots (tillers) are located only at the most basal nodes of the plant (crown) (Fig 1B) [17,18]. After transition from vegetative to reproductive development, the grass apex irreversibly differentiates into the inflorescence. Domestication of different crop species has resulted in cultivars with altered shoot architecture. For example, *TEOSINTE BRANCHED 1* (*TB1*) gene, a TCP transcription factor and homolog of the *Arabidopsis thaliana* gene *BRANCHED 1* (*BRC1*), has been identified as a major domestication related gene in maize [19]. In cultivated maize, a natural overexpressing allele was introgressed into the breeding germplasm to reduce tillering as an adaptation to agricultural cultivation [19]. A homolog of *TB1*, *Intermedium-c* (*Int-c*) was also targeted in barley during domestication to increased lateral spikelet development. In addition, the *int-c* mutant has an increased tiller number at early developmental stages [20]. Similarly, the maize gene *BARREN STALK 1* (*BA1*), affects tiller and tassel development [21]. This exemplifies that genes involved in inflorescence branching can act on shoot branching. However, the pleiotropic effects of row type genes on shoot architecture are not straightforward: *int-c* and *int-m* are described as mutants with an increased tiller number while *int-b* and *int-l*.*81* (allelic to *low number of tillers 1*, *lnt1*) have a reduced tiller number [5,7,22]. It is therefore important to perform a comparative study on the pleiotropic effects of different row type genes on yield components.

To this end we recorded tiller number, seed number per spike, seed area and TGW of independent allelic mutants of *vrs3/int-a*, *int-b*, *int-c*, *vrs1/int-d*/*hex-v*,*vrs4*/*int-e*, *int-f*, *int-m*, *lnt1/int-l* and *absent lower laterals* (*als*). The latter is included since *als* mutants show irregularities in floret phyllotaxy and the gene is involved in secondary AM formation [23]. Given that tiller number is a dynamic trait, affected by the environment and development, we monitored tiller number at different developmental stages under greenhouse and outdoor conditions. Our data shows that row type genes affect the correlation between shoot and spike architecture either directly most likely via regulating the initiation and/or outgrowth of tillers or indirectly due to resource limitation. We conclude that the classification based on the macroscopic phenotypes presented here helps to understand and interpret the pleiotropic effects of row type genes on major yield components.

## Materials and Methods

### Plant material

Seeds of different row type mutants representing the loci (*vrs1/hex-v*/*int-d*, *vrs3*/*int-a*, *vrs4*/*int-e*, *int-b*, *int-c*, *int-f* and *int-m*) and their respective parental cultivars were obtained either from the Department of Agriculture of the United States of America (USDA) or from the Nordic Gene Bank (NordGen) (S1 Table). For clarity, different loci of the same gene will be designated with one name referring to the original locus with the allele name in brackets. In this study, we used the original mutants in the cultivars Bonus and Foma and Bowman backcross lines [24]. In addition, we analyzed different allelic mutations of the *Vrs1* gene originating from an EMS TILLING population in cv. Barke [25]; and two backcrosses (BC_3_) of six-rowed *Vrs1* mutants cv. Barke referred to as *vrs1*(*8408*) and *vrs1*(*11910*). Seed material of the TILLING mutants was kindly provided by S. Gottwald (University of Gießen) and N. Stein (IPK Gatersleben). Leaf material was collected from four individuals per genotype and freeze-dried. The freeze-dried material was ground and genomic DNA was extracted with the Qiagen BioSprint Robot (Progam: DNAPlant) and the respective Kit, according to the manufacturer’s recommendations (Qiagen, Hilden, Germany). As a control we verified the mutation in *vrs1*. Sequencing confirmed position and nature of the published mutations with exceptions of *vrs1*(*hex-v*.*3*) and *vrs1*(*hex-v*.*6*) (S2 Table).

### Outdoor experiment

Lines harboring different allelic variants of the *vrs* and *intermedium spike* loci (*vrs3/int-a*, *int-b*, *int-c*, *vrs1/hex-v/int-d*, *vrs4/int-e*, *int-f* and *int-m*) were grown outdoors and compared to their respective parental backgrounds. All phenotypes recorded were, when possible, confirmed in multiple independent allelic variants of the same locus in addition to backcrossed lines in the spring barley cultivar Bowman [24] (S1 Table). For *int-f*, *int-m* and *als*, only one allelic variant was available. The plants were sown in 96-well trays mid-February 2014, germinated in the greenhouse and then transferred outside (MPIPZ Cologne, Germany). After six weeks (end of March), plants were transferred to 12 L pots with one plant per pot, each filled with a custom-made peat and clay soil mixture (EinheitsErde^®^ ED73 Osmocote, Einheitserdewerke Werkverband e.V., Sinntal-Altengronau, Germany) containing a long term fertilizer. The pots were arranged in 22 rows with a distance of 1 m between rows where each row contained 54 pots with a distance of 10 cm. The entire experiment was divided into 4 blocks with at least 4 replicate plants per genotype within each block. To avoid edge effects, the plot was surrounded by border pots containing wild type barley plants.The plot was irrigated by a sprinkling robot and treated with additional fertilizer or pesticides when necessary. Tiller number was assessed at four different stages: (1) two weeks after transplantation (50 DAS), (2) flag leaf stage, (3) anthesis stage, and (4) at full maturity. Barke *vrs1* EMS TILLING lines were additionally also phenotyped at 56, 63 and 70 DAS. Flag leaf stage was recorded, when the flag leaf completely emerged and unfolded from the main shoot. At anthesis stage, at least one spike of the plant showed open florets with the anthers visibly pushed out. For *vrs4*.*k* tiller number at this stage was scored for only 10 plants instead of 16. This is due to the absence of a clear anthesis occurring in some individuals, which were therefore left out of the analysis. Total tiller number at full maturity was counted for all plants simultaneously mid of July 2014, two weeks before harvest. At this time point the number of grain bearing spikes was also recorded. At 92 DAS, pictures were taken from one representative plant per genotype. Plant height was measured (soil to base of topmost spike) at full maturity. All raw data obtained from the outdoor experiment has been added as S1 File.

### Seed analysis

The grain number per spike, seed size and shape and thousand grain weight (TGW) were measured using the MARVIN Seed Analyser (GTA Sensorik, Neubrandenburg, Germany).All seeds and spikes used in the analysis of the seed parameters were derived from plants grown outdoors. With exception of *int-b*.*6*, all measurements were performed on at least 3 spikes from 4 individual plants per genotype. For *int-b*.*6*, only 2 spikes of 4 individual plants were scored due to the low tiller number. The frequency distribution of seed size was calculated and plotted against the average number of seeds per spike. The TGW was measured for all mutant and wild type lines based on the total number of seeds of the 3 spikes from 4 separate plants per genotype. In addition, the TGW for *vrs3*, *int-b*.*75*, *int-c*, *vrs1*, *vrs4*, *int-m* and *int-f* and wild type genotypes, but not for the low tillering *int-b*.*3*, *int-b*.*6*, *als* and *lnt* mutants, was measured from three different plants per genotype using 4x5 mL of seeds (240–480 seeds in total). No significant differences were observed when comparing the TGW extrapolated from the seed weight per spike with the TGW obtained from measuring 240–280 seeds per plant (S3 Table). All raw seed parameters measured are included as S2 File.

### Greenhouse experiments

Variation in tiller number of mutant and wild type genotypes was also scored in a controlled greenhouse under long day (LD) conditions (16h, 22°C day; 8h, 18°C night). Tiller number at full maturity was recorded for different allelic row type mutants (*vrs3/int-a*, *int-b*, *int-c*, *vrs1/hex-v/int-d*, *vrs4/int-e*, *int-f* and *int-m*). Due to spatial limitations in the greenhouse, the mutants and respective wild type genotypes were grown in separate experiments in 1.3 or 4 L pots. The mutant lines *int-c*.*25* and *-c*.*29* in Foma, *int-c*.*5* in Bonus, *int-f*.*19* in Foma and all other row type mutant lines backcrossed to Bowman were scored for tiller number twice per week starting from 7 days after emergence (DAE) until the age of four weeks and once per week thereafter until the age of 13 weeks (n = 10 plants, grown in 1.3 L pots). The flag leaf stage was recorded as in the outdoor experiment. Pictures were taken from three representative plants per genotype at 32 DAE.

For staging the development of the shoot apical meristem (SAM) of all mutants in Bowman background and *int-f* in Foma background, seeds were sown in 96-cell “Mini Tray” (Einheitserde^®^) and kept at 4°C for 3 days after which they were germinated under LD conditions (16h, 22°C day; 8h, 18°C night). The apex development of 3–5 plants was recorded at 12, 18 and 22 DAE in three independent biological replicates. The SAM was visualized using a stereo microscope (MZ FLIII, Leica) and the developmental stage was determined using the quantitative scale introduced by Waddington et al. [26]. *als* was not included in this analysis due to seed limitations.

### Data analysis

Statistical analyses were performed using the statistical software R (http://www.r-project.org/) or SAS version 9.1 (SAS Institute 2003). Differences between wild type and mutant genotypes were determined using a student’s t-test or a one-way ANOVA combined with a Tukey HSD for multiple comparison for the outdoor and greenhouse experiments separately. The mutant genotypes were classified into groups based on the relative performance of each mutant line according to the following equation:

$$\text{relative}\;\text{performance}\;=\;\frac{mut-\;\text{wt}}{\text{wt}}$$

in which “*mut*” denotes the mutant line and “wt” wild type. Calculations were performed on the trait averages for each mutant line. The relative performance values were used to cluster the different mutants using a hierarchical cluster analysis (HCL) [27]. The HCL analysis was done using the MeV multiple experiment viewer (http://www.tm4.org/mev.html), with default settings that apply a Pearson correlation as distance measure and average linking for clustering.

Significant effects of the mutant locus and environment (greenhouse or outdoors) on variation in tillering were calculated using the following generalized linear model (GLM) programmed in SAS:

y=gene (allele) + environment + gene*environment + gene(allele)*environment

Were gene is the fixed effect of the mutant locus; gene(allele) the fixed effect of the allele nested within the mutant locus; environment the fixed effect of the environment (greenhouse vs. outdoor), and the interaction effects between the gene and environment and between the environment and the mutant allele nested within the mutant locus. We also tested for significant block effects in the outdoor experiment in an initial GLM model. However, since the block showed no significant effects on tillering, this factor was removed from the final model. In order to test if the mutant locus exhibited consistent effects on tillering across the mutant alleles and environments, least square means were calculated within the GLM procedure for the effect of the mutant locus on tillering under greenhouse and outdoor conditions separately.

## Results

### Trade-off between seed number per spike and seed size

To decipher the effects of row type genes on seed setting (fertility) and seed size, we analyzed the seed number per spike, TGW and seed shape parameters (width, length and area) for different row type mutants. To compare the phenotype of the different row type mutants directly, backcrossed lines in the spring barley cultivar Bowman were used [24] in addition to the original mutants [5] and allelic series of *vrs1* mutants in cv. Barke identified by EMS TILLING [25]. When available, multiple independent allelic variants of the same locus were used to test if pleiotropic effects were locus specific (S1 Table). The mutant *vrs3*(*int-a*.*31*) in Foma background was removed from the analysis since this mutant exhibited pre-harvest sprouting and therefore no reliable measurements on the seed size and TGW could be performed. None of the other row type mutants showed pre-harvest sprouting.

**Fig 2:**
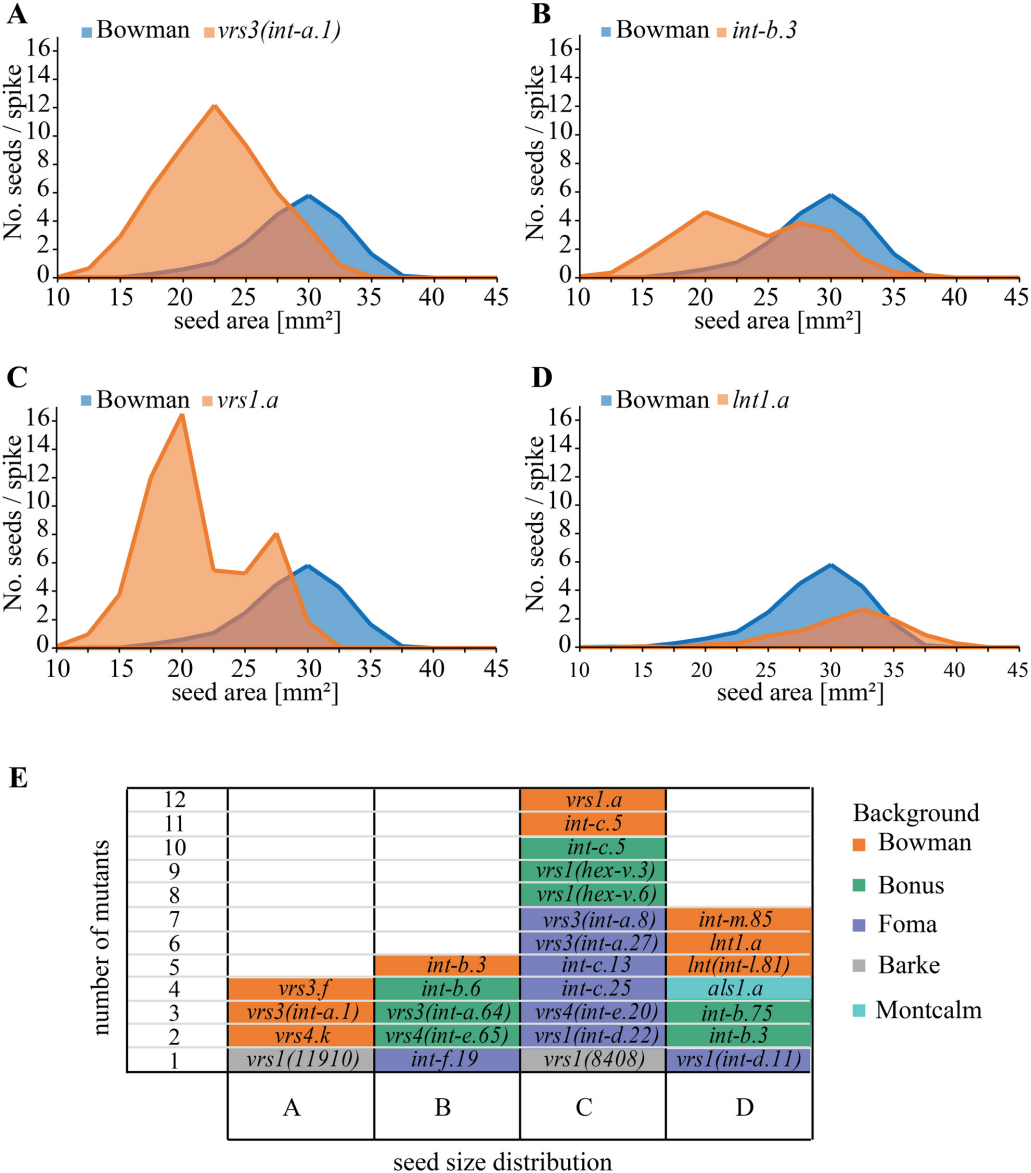
Distribution of seed size is altered in row type mutants. Distribution of seed area ranging from 10–45 mm^2^ plotted against the average seed number per spike (n≥8 spikes). The spikes used to obtain the frequency distribution of seed size and average seed number per spike were derived from plants grown under outdoor conditions. Fig A-D represents typical seed size distributions measured in all genotypes and backgrounds. (**A**) Bell-shaped distribution with higher seed number and variance and lower average: e.g. *vrs3(int-a*.*1)*; (**B**) bimodal distribution with one peak at a size similar to wild type and one peak at a smaller size, both peaks similar in height: e.g. *int-b*.*3*; (**C**) bimodal distribution with one peak at a size similar to the wild type and one peak at a smaller size accounting for approximately twice the number of seeds of the larger size peak: e.g. *vrs1*.*a*; and (**D**) seed size distribution similar to wild type and a reduced seed number per spike: e.g. *lnt1*.*a*. (**E**) Number of different row type mutants grouped based on the distribution in seed size as described in **A-D**.

The highest increase in seed number per spike is found for six-rowed mutants: *vrs1*(*11910*), *vrs1*(*8408*), *vrs1*.*a*, *vrs1*(*hex-v*.*3*), *vrs1*(*hex-v*.*6*), *vrs4*.*k* and for *vrs3*(*int-a*.*1*) in Bowman background. These mutations increase the seed number per spike on average 2.5-fold. In our analysis, the majority of the row type mutants exhibit an increase in the seed number per spike and a reduction in the TGW (Table 1). This indicates a trade-off between the seed number per spike and seed size. Seed size of the two-rowed parental cultivars Barke, Bonus, Bowman or Foma show a bell-shaped distribution. When compared to these, an increase in seed number per spike results in either an overall reduction of seed size or in less homogenous sized seeds on the same spike (Fig 2A–2C, S1, S2, S3 and S4 Figs). In the *vrs1*.*a* allele about 2/3 of the seeds exhibit a size between 15–22.5 mm while 1/3 of the seeds have a size between 22.5–30 mm, resulting in a bimodal distribution (Fig 2C). In contrast to the *vrs1*.*a* allele, *int-m*.*85* (Bowman), *lnt1* (Bowman) and *als1*.*a* (Montcalm) exhibit a significant reduction of the seed number per spike and a reduction in TGW (Table 1). In these mutants, the seed number per spike and TGW thus shows a positive correlation (S5 Fig). The *int-b* mutants exhibit a slight reduction in seed number per spike and a significant reduction in TGW. The reduction of seed number per spike is caused by different spike phenotypes. The lateral spikelets of the *vrs1*(*int-d*.*11*) mutant, which harbors a mutation within the C-terminus of *Vrs1*, do not set seed. Therefore, the spikes of the *vrs1*(*int-d*.*11*) mutant remain effectively two-rowed and do not show a significant reduction of seed number per spike or TGW. The *int-m*.*85* have very short spikes, while *lnt1*, *int-b* and *als* mutants display an irregular seed set [22,23]. In addition, the seed size distribution of these lines is only marginally different from the respective two-rowed wild type backgrounds (Fig 2D, S1, S2, S3 and S4 Figs). In conclusion, the majority of the row type mutants exhibit an increased seed number per spike and an overall reduction in seed weight (Fig 2E, Table 1, S5 Fig).

**Table 1:** Average seed number per spike and thousand grain weight (TGW) of row type mutants.

			seeds per spike		TGW	
background	locus	allele	avg^1^	difference^2^ (%)	avg (g)^1^	difference^2^ (%)
**Bowman**			20.8±2.2^a^		57.2±5.1^a^	
	*vrs3*	*int-a*.*1*	51.3±5.2^b^	147	39.6±2.5^b^	-31
	*vrs3*	*vrs3*.*f*	43.3±5.8^bc^	108	38.6±1.9^b^	-33
	*int-b*	*int-b*.*3*	25.6±10.8^a^	23	43.7±6.6^b^	-24
	*int-c*	*int-c*.*5*	44.7±8.6^c^	115	37.7±3.7^b^	-34
	*vrs1*	*vrs1*.*a*	54.1±5.2^b^	160	37.0±2.7^b^	-35
	*vrs4*	*vrs4*.*k*	56.8±10.6^b^	173	41.7±3.6^b^	-27
	*int-m*	*int-m*.*85*	8.7±2.1^d^	-58	53.5±4.6^ab^	-6
	*lnt1*	*int-l*.*81*	13.1±2.9^d^	-37	47.0±9.5^b^	-18
	*lnt1*	*lnt1*.*a*	10.1±4.7^d^	-51	52.5±6.4^ab^	-8
**Bonus**			28.7±2.7^a^		63.8±4.2^a^	
	*vrs3*	*int-a*.*64*	39.6±6.3^b^	38	48.1±3.7^b^	-25
	*int-b*	*int-b*.*3*	20.8±9.1^a^	-27	40.7±7.4^c^	-36
	*int-b*	*int-b*.*6*	21.1±7.0^a^	-26	39.2±4.9^c^	-39
	*int-b*	*int-b*.*75*	20.8±11.8^a^	-28	44.6±7.4^bc^	-30
	*int-c*	*int-c*.*5*	58.5±11.0^c^	104	47.4±4.2^b^	-26
	*vrs1*	*hex-v*.*3*	73.9±11.1^d^	158	40.3±3.3^c^	-37
	*vrs1*	*hex-v*.*6*	68.8±9.6^d^	140	40.5±4.5^c^	-37
	*vrs4*	*int-e*.*65*	38.8±6.4^b^	35	53.7±3.7^d^	-16
**Foma**			29.6±2.4^a^		54.6±3.4^a^	
	*vrs3*	*int-a*.*8*	43.3±9.2^ab^	46	47.0±5.7^b^	-14
	*vrs3*	*int-a*.*27*	49.3±6.8^b^	66	48.0±3.2^b^	-12
	*int-c*	*int-c*.*13*	52.8±9.3^b^	78	44.0±4.2^b^	-20
	*int-c*	*int-c*.*25*	54.1±11.8^b^	83	47.2±4.4^b^	-14
	*vrs1*	*int-d*.*11*	25.9±2.2^a^	-13	54.8±3.5^a^	0
	*vrs1*	*int-d*.*22*	61.8±11.5^c^	109	32.2±3.4^c^	-41
	*vrs4*	*int-e*.*20*	44.3±7.8^b^	49	43.8±3.5^b^	-20
	*int-f*	*int-f*.*19*	37.4±6.7^ab^	26	52.4±4.6^a^	-4
**Barke**			27.0±2.6^a^		60.1±5.9^a^	
	*vrs1*	*8408* BC_3_	66.8±12.1^b^	148	37.4±5.0^b^	-38
	*vrs1*	*11910* BC_3_	66.3±9.6^b^	146	40.7±7.0^b^	-32
**Montcalm**			61.0±8.3^a^		54.2±3.5^a^	
	*als*	*als1*.*a*	24.6±4.3^b^	-60	34.8±6.7^b^	-42

^1^average values with standard deviations (n ≥ 8 spikes, derived from plants grown outdoors).

Letters indicate differences between the mutants based on a one-way ANOVA (*p* ≤ 0.05), using a Tukey HSD for multiple comparison.

^2^difference compared to the respective wild type.

### Row type genes have pleiotropic effects on tillering

**Fig 3:**
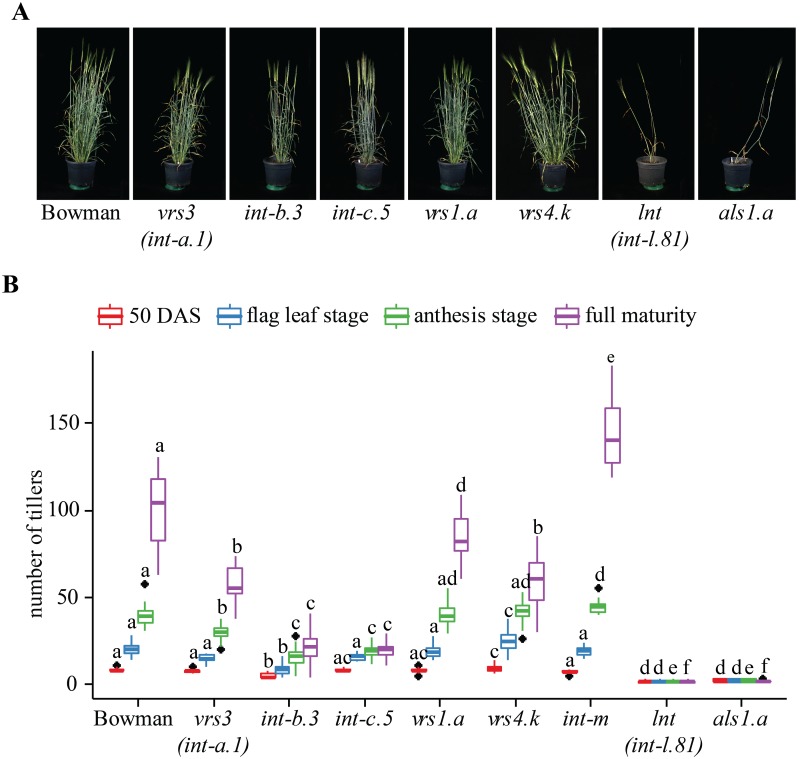
Pleiotropic effects of mutations in different row type genes on tiller number cv. Bowman. Plants were grown under outdoor conditions. (**A**) Photographs of one representative plant per genotype were taken at 92 DAS. No pictures were taken of *int-m*.*85* mutants, because no tillering phenotype was visible at this point. (**B**) Tiller number was assessed two weeks after transplantation (50 days after sowing), at flag leaf stage, at anthesis stage and at full maturity. For all measurements n ≥ 10 plants, error bars ± SD. Letters a-f indicate significant differences (*p*≤ 0.05), determined with a one-way ANOVA using a Tukey HSD as post hoc test. Significant differences between the lines are calculated for each time point separately.

To assess the effect of row type genes on tillering, the tiller number of different row type mutants was analyzed at 50 days after sowing (DAS), at the flag leaf and anthesis stage, and before harvest of dry plants (full maturity) (Fig 3, S6, S7 and S8 Figs, S4 Table, S1 File). To reduce effects on the tiller number due to spatial limitation (e.g. pot size), competition or nutrient shortage, plants were grown individually in 12 L pots under semi-controlled outdoor conditions. In addition, plants were randomized within the plot to avoid differences due to spatial effects.

**Fig 4:**
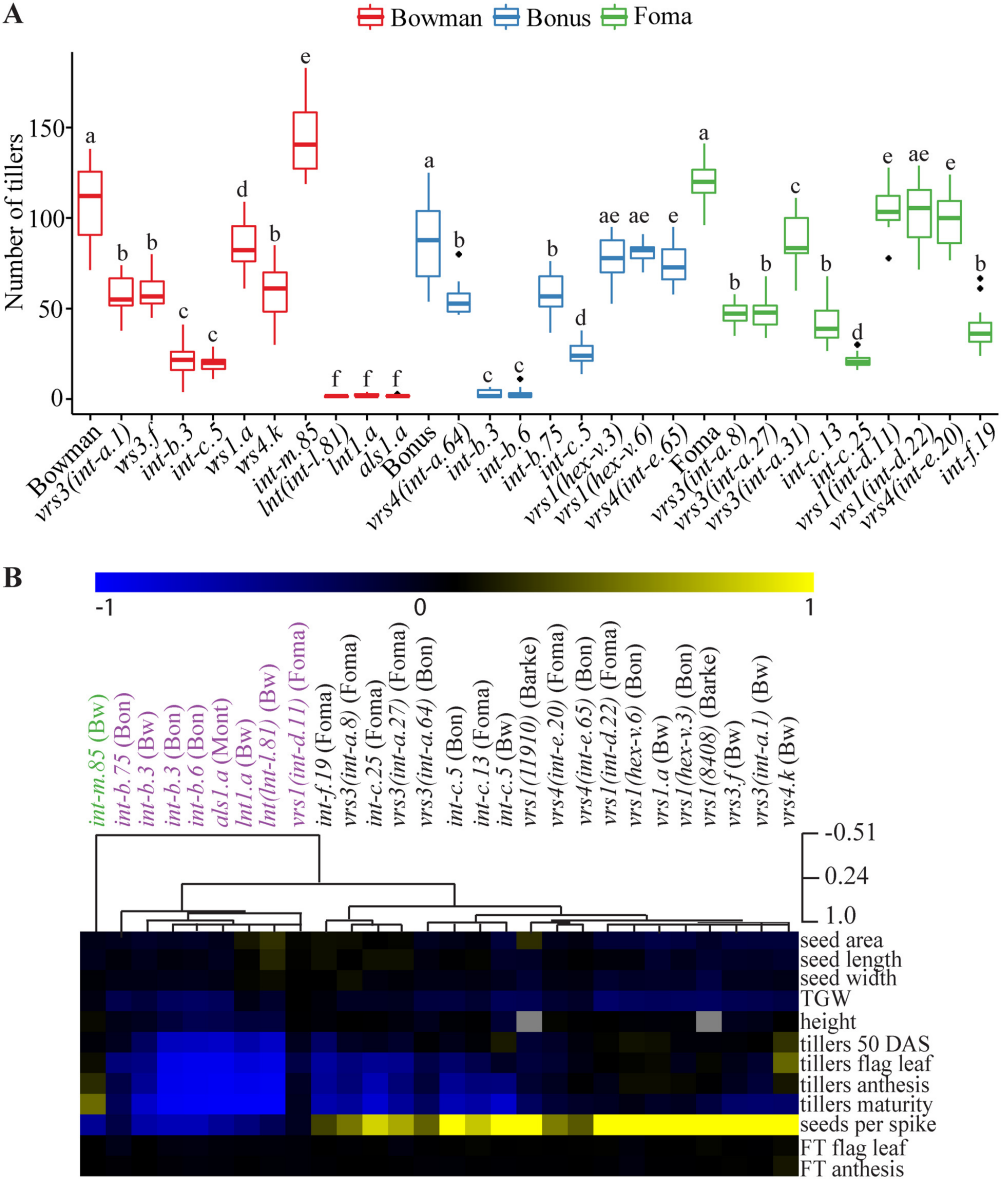
Row type mutants cluster according to their trait phenotypes. (**A**) Boxplots of tiller number at full maturity of all mutants in backgrounds Bowman (red), Bonus (blue) and Foma (green) tested under outdoor conditions (n ≥ 10 plants). Significant differences (*p*≤ 0.05) between the mutant and wild type parent were calculated using a one-way ANOVA with a Tukey HSD as post-hoc test. (**B**) Hierarchical cluster analysis (HCL) of the relative performance of different row type mutants compared to their respective backgrounds, Bowman (Bw), Bonus (Bon), Foma, Barke and Montcalm (Mont). All trait data used in the HCL analysis was obtained from plants grown outdoors. Relative performances were analyzed with a Pearson correlation and the mutants were clustered accordingly. The clustering shows one outgroup (green) and two main groups color-coded in black and purple.

All row type mutants in Bowman background show a reduced tiller number at full maturity compared to Bowman, with the exception of *int-m*.*85* which exhibit significantly more tillers than Bowman at maturity (Fig 3). In addition, multiple allelic variants of *lnt1*, *int-b*, and the single mutant allele of *int-f* (*int-f*.*19* in Foma) and *als* (*als1*.*a* in Montcalm and Bowman backcross), respectively, have a low tiller number at all developmental stages, whereas *int-c* and *vrs3* show a reduction in tillering only after the flag leaf stage (Fig 3, S6, S7 and S8 Figs, S4 Table). The *vrs1*.*a* and *vrs4*.*k* mutants in Bowman background only show a decrease in tiller number at full maturity. One exception to this is *vrs1*(*11910*) in Barke background, which exhibits a lower tiller number over the whole development. None of the other *vrs1* mutants shows a reduction in tiller number before maturity, suggesting that a background mutation rather than the *vrs1* mutation itself caused the tiller phenotype in *vrs1*(*11910*). Notable differences between allelic variants and backgrounds are observed (S6, S7 and S8 Figs, S4 Table). For example, *vrs1*(*hex-v*.*3*) and *vrs1*(*hex-v*.*6*), two six-rowed allelic variants of *vrs1* in Bonus background, do not show a significant reduction in tiller number (Fig 4A, S6 Fig, S4 Table). In contrast, *vrs1*(*int-d*.*11*) and *vrs1*(*int-d*.*22*), two *vrs1* mutants with *intermedium spike* phenotypes, exhibit a reduced tiller number at full maturity (Fig 4A, S7 Fig, S4 Table). In addition, *int-b*.*3* and *int-b*.*6* in Bonus background display a more severe reduction in tiller number when compared to the *int-b*.*75* allele in the same background (Fig 4A). Interestingly, the reduction in tiller number in *int-b*.*3* is less severe in the Bowman backcross (Fig 4A), suggesting an effect of background variation between Bonus and Bowman or the presence of additional mutations in the original line.

As flowering time can affect shoot branching by influencing the length of vegetative development [28–30], we also compared flowering time and apex development between the different mutants and respective wild type genotypes. The row type mutants do not show major differences in flowering time or apex development when compared to their respective wild type backgrounds (S4 Table, S9 Fig). In addition, plant height was measured at full maturity. The low tillering mutants *lnt1*.*a*, *als1*.*a* and *int-b* are significantly shorter compared to their respective parents. No consistent height phenotype is observed for any of the other row type mutants (S4 Table). These results indicate that row type genes mainly affect seed parameters and tiller number, but neither flowering time nor plant height. Variation within these traits is not only dependent on the allelic variants but also on the genetic background of the parental lines. To compare the effects of mutant loci across different backgrounds, the relative phenotypic performance of each mutant was calculated for all traits compared to their respective parent. Hierarchical clustering [26] of the relative performance shows that the row type mutants cluster into two main groups (Fig 4B). Mutants in group 1, which contain all alleles of *vrs3*, *int-c*, *int-f*, *vrs4*, and *vrs1* (with exception of *vrs1*(*int-d*.*11*)) show a negative correlation between tillering and seed number per spike. Group 1 can be subdivided into two subgroups: (a) low tillering already before maturity (*vrs3*,*int-c*, *int-f*); and (b) low tillering only at maturity (*vrs1*,*vrs4*). Group 2 encompasses all alleles of *als*, *lnt1*, *int-b*, and *vrs1*(*int-d*.*11*). These mutants display reduced tillering and seed number per spike. Within group 2 *vrs1*(*int-d*.*11*) and *int-b*.*75* form a separate cluster, as they are characterized by a less severe tillering phenotype when compared to the other mutants in group 2. The *int-m*.*85* mutant, which forms an outgroup, is the only row type mutant with an increased tiller number at full maturity and reduced number of seeds under outdoor conditions.

To test if the mutant loci exhibit a consistent effect on tillering across environments, variation in tillering at maturity was scored outdoors and in a controlled greenhouse. Under greenhouse conditions, plants have less tillers when compared to those grown in the outdoor experiment (S10 Fig). Indeed, the analysis of variance confirmed that the environment has a significant effect on tillering (S5 Table). The effect of each mutant locus on tillering is stronger than that of the environment for the majority of genotypes. With exception of *vrs1* and *int-m*, all mutant loci cause a consistent reduction in tiller number in the greenhouse and outdoor experiment (Table 2). The reduction and increase of tiller number in *vrs1* and *int-m*, respectively, is only significant under outdoor conditions (Table 2). The effects of the mutant loci on tillering are consistent over different mutant alleles (S5 Table, S10 Fig).

**Table 2:** Least mean square values for row type mutants under greenhouse and outdoor conditions.

gene	genotype	LSMEAN greenhouse		LSMEAN outdoors	
***vrs3***	mutant	13	**	59	***
	wt	22		103	
***int-b***	mutant	11	**	24	***
	wt	18		94	
***int-c***	mutant	16	***	22	***
	wt	30		103	
***vrs1***	mutant	14	n.s.	86	***
	wt	16		108	
***vrs4***	mutant	11	***	67	***
	wt	18		95	
***int-f***	mutant	17	***	26	***
	wt	28		111	
***int-m***	mutant	13	n.s.	144	***
	wt	10		101	
***lnt1***	mutant	3	***	2	***
	wt	11		101	
***als***	mutant	3	*	2	***
	wt	11		77	

Stars (*) indicate significant differences between mutant and wild type,

* p-value < 0.05,

**p-value <0.01,

***p-value <0.001.

Taken together, the majority of row type mutants show consistent effects on tillering across different environments, allelic variants and genetic backgrounds. Mutant loci which affect tillering only at later developmental stages, such as *vrs1* and *int-m*, have no significant effects on tillering when plants are grown in the greenhouse. In addition, the cluster analysis classifies the row type mutants based on their macroscopic phenotype into three groups; mutants in group 1 have a negative correlation between tillering and seed number per spike already after flag leaf stage (group 1a) or only at full maturity (group 1b), while mutants in group 2 exhibit a positive correlation between these traits

### Tillering phenotype of *int-c* changes during development

**Fig 5:**
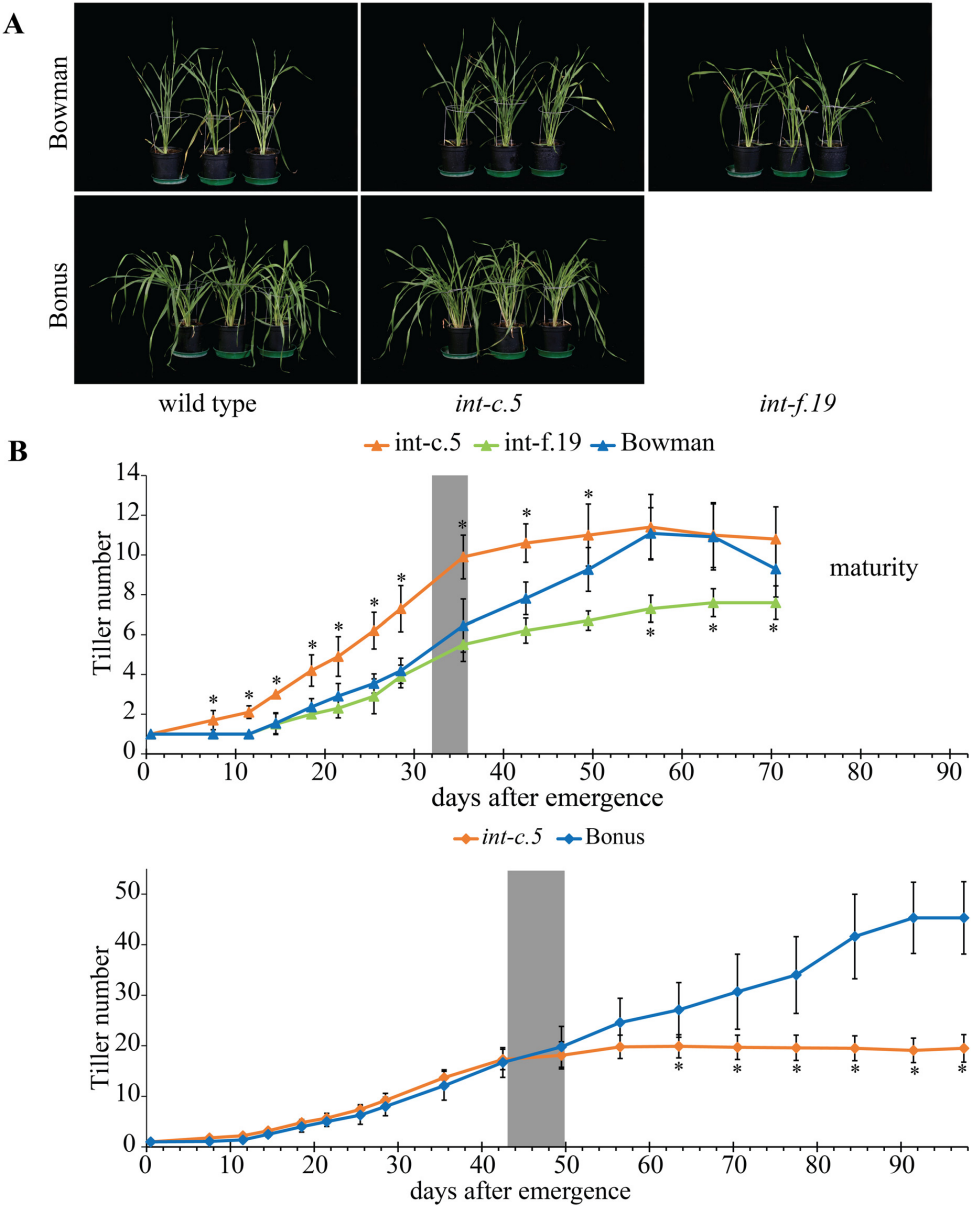
*int-c* mutants are high tillering only at early developmental stages. Ten biological replicates per genotype were grown in a controlled greenhouse under long day (LD) conditions (16 h light). (**A**) Pictures of three representative plants per genotype at 32 DAE. (**B**) *int-c*.*5* and *int-f*.*19* in Bowman background together with Bowman. (**C**) The original *int-c*.*5* mutant with its parent Bonus. The gray area indicates the time period when plants reached flag leaf stage. For all measurements n = 10 plants, error bars ± SD. * indicate significant differences between the mutants and the background (*p* ≤ 0.05) determined with a one-way ANOVA using a Tukey HSD as post hoc test.

Mutants of *int-c* (*HvTB1*) have been described as high tillering [20], however in our analysis no such increase in tiller number was observed. In fact, the tiller number of *int-c* mutants is significantly reduced compared to the wild type parents at flag leaf stage and later stages of development (Fig 3, S6, S7 and S8 Figs, S4 Table). This observation appears to be in contrast with a study which reported an increased tiller number in *int-c* mutants at an early developmental stage [20]. We therefore recorded tillering of *int-c* over the entire life span of the plant under greenhouse conditions. Three independent mutants of *int-c* were selected: *int-c*.*25* and *-c*.*29* in Foma background and *int-c*.*5* in Bonus and Bowman backgrounds. In addition, *int-f*.*19* in Foma and Bowman background was selected as another low tillering row type mutant which showed a reduction in tiller number over the whole development (Fig 5, S11 Fig). Interestingly, *int-c*.*5* mutants in Bonus background do not exhibit an increase in tiller number while the same mutation backcrossed into Bowman background shows a significant increase in tiller number when compared to the respective parental line until 56 DAE. However, *int-c*.*5* in Bonus background has a significant reduction in tiller number after 63 DAE (Fig 5). The parental lines Bowman, Bonus and Foma form new tillers until full maturity while all *int-c* mutants tested cease formation of new tillers after the flag leaf stage under both outdoor and greenhouse conditions (Fig 5, S11 Fig). As a consequence, the wild type plants have significantly more tillers at maturity when compared to flag leaf stage, while *int-c* mutants do not (S6 Table). No differences were observed in tiller number at full maturity between Bowman and *int-c*.*5* under greenhouse conditions while in the outdoor experiment *int-c*.*5* in Bowman showed a significant reduction at full maturity. When grown in the greenhouse, Bowman and *int-c*.*5* in Bowman background reached flag leaf stage after only five weeks (32 to 36 days after emergence) while in the outdoor experiment this stage was reached after 62 DAS. The fast development under greenhouse versus outdoor conditions and between Bowman and Bonus/Foma might cause the variation observed in relative performance of *int-c* mutants in different backgrounds/conditions.

To evaluate if any of the other row type mutants show a consistent increase in tiller number at early development but a reduction after flag leaf stage, we analyzed the tiller number over their lifespans. For comparison, we used only row type mutants backcrossed into the Bowman parent grown under greenhouse conditions. However, none of the other row type mutants shows a similar change in tillering behavior as was seen in *int-c* (S12 Fig, S7 Table). Taken together, our data shows that when compared to the wild type, the tiller number of *int-c* is increased at early developmental stages and reduced at later stages, as the duration of tiller formation is reduced by the *int-c* mutation.

## Discussion

Shoot and spike architecture are two important adaptive traits in barley. Our results show that an increase in the seed number per spike as observed in the *intermedium* and six-rowed mutants causes an overall reduction in seed size. In addition, variation in seed size is increased in the row type mutants due to the size difference between central and lateral spikelets. In the *vrs1* and *int-c* mutants the seed area shows a bimodal distribution with a 2:1 ratio. In line with this, grains of the lateral florets are generally smaller when compared to the central florets [11]. *Vrs1*, *Int-c* and other *Vrs* loci have been reported to influence seed weight and shape parameters in a doubled haploid population derived from a cross between two-rowed cv. Harrington and six-rowed cv. Morex. *Vrs1* was found to be the major locus affecting TGW and all seed size and shape parameters measured [11]. Similarly, in our analysis *vrs1* causes the largest increase in seed number per spike and the most severe reduction in TGW when compared to the other row type mutants. The increase in the seed number per spike was likely the reason for the introduction of the *vrs1* allele into the barley gene pool during domestication [8]. In addition, six-rowed cultivars generally harbour an allele of *Int-c* (*Int-c*.*a*) which increases lateral seed size compared to the allele present in two-rowed cultivars (*Int-c*.*b*) [11,20]. Strong variation in seed size is generally considered unfavorable for malting barley which are therefore most commonly grown in Europe as two-rowed cultivars [11,31].

While the majority of the row type mutants shows a negative correlation between seed number and TGW, *int-m*.*85*, *lnt1*, *als* and several alleles of *int-b* display a reduction in seed number and in seed size. A positive correlation between seed number and seed size in these mutants suggests that the causative mutations negatively affect both seed size and seed number. These findings indicate that pleiotropic effects between yield parameters are not only caused by competition of different plant organs for limited resources, but that row type genes may control development of different meristematic tissues. We therefore analyzed the effects of row type genes not only on spike, but also on shoot branching. Indeed, we observed that row type mutants are affected in tillering. As observed for the correlations between seed number and size, we identified mutants with negative and positive correlations between seed number and tiller number. Based on these correlations, we classified the row type mutants into two main groups: (1) mutants with increased seed number per spike and low tillering; and (2) mutants with a reduction in seed and tiller number. Row type mutants in group 1 show an increase in inflorescence branching (seed number per spike) while the shoot branching (tiller number) is reduced after flag leaf stage (group 1a) or only at full maturity (group 1b). A similar effect of increased inflorescence branching and decreased tillering was reported for certain alleles of rice *SQUAMOSA BINDING PROTEIN-LIKE 14* (*OsSPL14*), which were found to be positively associated with desirable traits underlying a quantitative trait locus (QTL) termed Ideal Plant Architecture (IPL) or Wealthy Farmer’s Panicle (WFP). Alleles of *OsSPL14* with higher expression levels cause a reduction in shoot branching, but also stronger culms and increased panicle branching leading to higher yield. Knockdown of *OsSPL14* results in more tillers and a reduction in plant height, culm diameter, panicle branches and grain number [32,33]. Furthermore, the wheat *tiller inhibition* (*tin*) mutant displays larger spikes and a higher grain number per spikelet and a low tiller number. The spike phenotype can be phenocopied by removal of tillers shortly after initiation in wild type plants [34]. This exemplifies that different plant organs can compete for the same resources, resulting in a reduction of tiller number which correlates with an increase in seed number per spike. We observed that mutants of group 1b (*vrs1*, *vrs4*) with a high seed number per spike exhibit a reduced tiller number at maturity, but not at earlier stages of development. No tillering phenotype was observed in *vrs1* mutants in the greenhouse experiment or until the plant reached full maturity in the outdoor experiment suggesting that *vrs1* does not directly affect the initiation and outgrowth of axillary buds. This is supported by the finding that, unlike its paralog *HvHox2*, which is expressed abundantly in all tissues, *Vrs1* is expressed almost exclusively in developing spikes [35]. Therefore, the observed reduction in tiller number is likely caused by resource limitations. Mutants of *vrs4* show a similar trend, with no reduction in tiller number until the plant reached full maturity. In line with this, *Vrs4* was reported to act upstream of *Vrs1* in the same pathway [9], suggesting that this signaling cascade does not directly influence shoot branching. In contrast to *vrs1* and *vrs4*, *int-c* mutants show an increase in tiller number at early developmental stages (this work and [20]), suggesting a direct effect of this gene on tillering. A similar increase in tiller number has been observed in loss of function mutations in maize *TB1* or rice *FINE CULM 1* (*FC1*), two homologs of *int-c* [20,36,37]. *TB1* and *FC1* directly affect tiller number by suppressing the outgrowth of lateral buds. In the *fc1* mutant it has been demonstrated that the increase in tiller number is due to reduced dormancy of the buds at the prophyll node, the most basal node of the tiller [36]. *Int-c* in barley may affect bud outgrowth in a similar way by preventing the outgrowth of axillary buds. Interestingly, our data shows that *int-c* mutants cease to grow out new tillers after reaching flag leaf stage which causes the observed reduction in tiller number at late developmental stage. This indicates that the phenotype of *int-c* mutants differs from its homologs *tb1* or *fc1* which exhibit an increase in tiller number throughout all developmental stages [36,37]. None of the other row type mutants exhibit an increase in tiller number at very early developmental stages. In fact, *vrs3* mutants which are also clustered in group 1a have significant reduction in tiller numbers starting from flag leaf stage on.

The *als*, *lnt1* and *int-b* mutants in group 2 show an even stronger reduction in tillering throughout their development. This is in line with previous observations that *lnt* and *als* successfully initiate primary AMs, but fail to generate secondary AMs [22,23]. Despite a similar phenotype, *lnt1* and *int-b* likely act in different pathways as double mutants do not produce any tillers [22]. As *als*, *lnt1*, and *int-b* exhibit a strong decrease in seed number, seed size and tiller number, the causative mutations may directly initiate the formation or outgrowth of different meristematic tissues in barley. It has been shown before that tillering genes also modify seed number. For example, the rice gene *MONOCULM 1* (*MOC1*), a homolog of the *A*. *thaliana* boundary gene *LATERAL SUPPRESSOR* (*LAS*), affects shoot as well as inflorescence branching [38].

In line with this, we propose that row type mutations in barley directly affect tiller initiation or outgrowth as suggested by the strong effects on tillering at early developmental stages. One exception to this are mutations in *vrs1* and *vrs4* which cause a reduction in tillering only at maturity likely due to resource limitations.

## Conclusions

Shoot architecture is crucial for crop yield in cereals. We have shown that genes involved in development of the branched inflorescence architecture of the grasses also control seed size and shoot branching. Our results indicate that correlations between shoot and spike architecture are due to a) competition between different sink organs for limited assimilates or b) the direct involvement of row type genes in the initiation and growth control of different plant organs, seeds and tillers. We thus speculate that the same regulatory genes or modules may control the development of different meristematic structures and organs in plants. Understanding how these genes are regulated and in turn control downstream targets in different plant organs is important to improve yield by modifying shoot and spike architecture. The results presented here provide a starting point for such studies.

## Supporting Information

S1 Fig Row type mutants have altered seed area compared to cv.s Bowman and Montcalm. Graphs show the distribution of the seed area ranging between 10–45 mm^2^, n ≥ 15 spikes. (**A**) *int-c*.*5*, (**B**) *vrs4*.*k*, and (**C**) *int-m*.*85* in Bowman background. (**D**) *als1*.*a* compared to Montcalm. All parameters were derived from plants grown outdoors.
(TIF)

S2 Fig Row type mutants have altered seed area compared to cv. Bonus. Graphs show distribution of the seed area ranging between 10–45 mm^2^, n ≥ 8 spikes. (**A**) *vrs3*(*int-a*.*64*), (**B**) *int-b*.*3*, (**C**), *int-b*.*6*, (**D**) *int-b*.*75*, (**E**) *int-c*.*5*, (**F**) *vrs1*(*hex-v*.*3*), (**G**) *vrs1*(*hex-v*.*6*), and (**H**) *vrs4*(*int-e*.*65*). All parameters were derived from plants grown outdoors.
(TIF)

S3 Fig Row type mutants have altered seed area compared to cv. Foma. Graphs show the distribution of the seed area ranging between 10–45 mm^2^, n ≥ 8 spikes. (**A**) *vrs3*(*int-a*.*8*), (**B**) *vrs3*(*int-a*.*27*), (**C**) *int-c*.*13*, (**D**) *int-c*.*25*, (**E**) *vrs1*(*int-d*.*11*), (**F**) *vrs1*(*int-d*.*22*), (**G**) *vrs4*(*int-e*.*20*), and (**H**) *int-f*.*19*. All parameters were derived from plants grown outdoors.
(TIF)

S4 Fig Six-rowed *vrs1* mutants have altered seed area compared to cv. Barke. Graphs show the distribution of the seed area ranging between 10–45 mm^2^, n ≥ 8 spikes. (**A**) *vrs1*(*11910*) BC_3_S_3_, and (**B**) *vrs1*(*8408*) BC_3_S_3_. All parameters were derived from plants grown outdoors.
(TIF)

S5 Fig Row type mutants show positive or negative correlations between seed number per spike and TGW. (**A**) Correlation coefficients (r) of seed number per spike vs. TGW between row type mutants and their respective wild types. (**B-D**) Exemplary x-y scatterplots depicting data points from mutant and wild type and their respective correlation coefficients. All parameters were derived from plants grown outdoors.
(TIF)

S6 Fig Pleiotropic effects of mutations in different row type genes on tiller number cv. Bonus. Letters indicate groups of genotypes with significant differences from one another at the same time point determined by a one-way ANOVA (*p* ≤ 0.05). Tiller numbers shown in this graph are derived from plants grown outdoors.
(TIF)

S7 Fig Pleiotropic effects of mutations in different row type genes on tiller number cv. Foma. Letters indicate groups of genotypes with significant differences from one another at the same time point determined by a one-way ANOVA (*p* ≤ 0.05). Tiller numbers shown in this graph are derived from plants grown outdoors.
(TIF)

S8 Fig Pleiotropic effects of point mutations of *Vrs1* in Barke EMS TILLING lines. Letters indicate groups of genotypes with significant differences from one another at full maturity determined by a one-way ANOVA (*p* ≤ 0.05). Tiller numbers shown in this graph are derived from plants grown outdoors.
(TIF)

S9 Fig Development of the shoot apex of row type mutants compared to wild type under controlled greenhouse conditions. (**A**) *vrs3*, *int-b*, *int-c*, *vrs1*, *vrs4*, and *lnt1* in Bowman backcross; and (**B**) *int-f* in Foma background. Development was staged according to Waddington et al. [27]. Error bars represent standard deviation.
(TIF)

S10 Fig Average tiller number and relative performance of row type mutants in the outdoor experiment and under greenhouse conditions. Relative performance was calculated using the respective wild type growing under the same conditions. In case multiple experiments were performed under greenhouse conditions, only results from one representative experiment are shown. Results from all individual experiments can be found in S1 File. Stars (*) indicate a significant difference between the mutant and wild type determined by a one-way ANOVA (*p* ≤ 0.05).
(TIF)

S11 Fig Tillering of *int-c* mutants in cv. Foma. Ten biological replicates per genotype were grown in a controlled greenhouse under long day (LD) conditions. (**A**) Pictures of three representative plants per genotype were taken at 32 DAE. (**B**) This plot shows the tillering behavior of *int-c* and *-f* mutants from emergence until the age of 13 weeks after emergence. The gray area indicates the time period when plants reached flag leaf stage. The different genotypes within the same background did not differ significantly in flowering time. Bars indicate ± standard deviation. *Indicate significant difference (*p*≤ 0.05) when compared to Bowman background in *int-c*.*29* and *int-f*.*19* (**) or in *int-c*.*25*, *int-c*.*29* and *int-f*.*19* (***).
(TIF)

S12 Fig Row type mutants (except *int-c*) show no change in tillering behavior during devlopment. Ten biological replicates per genotype were grown in a controlled greenhouse under long day (LD) conditions. These plots show the tillering behavior of different row type mutants in Bowman background compared to Bowman until the age of ten weeks after emergence. (**A**) *vrs3*(*int-a*.*1*) and *vrs3*.*f*; (**B**) *int-b*.*3* and *int-m*.*85*; (**C**) *vrs1*.*a* and *vrs4*.*k*; and (**D**) *lnt1*(*int-l*.*81*), *lnt1*.*a* and *als1*.*a*. Bars indicate ± standard deviation. Statistical differences are shown in S5 Table.
(TIF)

S1 File Raw trait data obtained for the row type mutants under outdoor and greenhouse conditions. (XLSX)

S2 File Raw trait data seed parameters measured using the MARVIN analyser. (XLSX)

S1 Table Names and backgrounds of mutant alleles used in this study. (DOCX)

S2 Table Verification of the mutations in the *vrs1* gene. (DOCX)

S3 Table TGW extrapolated by measuring seed weight per spikes compared to measurements performed on 240–280 seeds. (XLSX)

S4 Table Tiller number, flowering time and plant height of row type mutants grown under outdoor conditions (XLSX)

S5 Table Genetic and environmental effect of row type mutants on tillering. (DOCX)

S6 Table *int-c* mutants do not change their tiller number significantly after flag leaf stage. (DOCX)

S7 Table Tillering behaviour of row type mutants in cv. Bowman during development when grown under greenhouse conditions. (XLSX)
