# Supplementary figures and images for: Mutations in Barley Row Type Genes Have Pleiotropic Effects on Shoot Branching
Source: PLoS One. 2015 Oct 14;10(10):e0140246. doi: 10.1371/journal.pone.0140246 (PMC4605766; doi:10.1371/journal.pone.0140246)

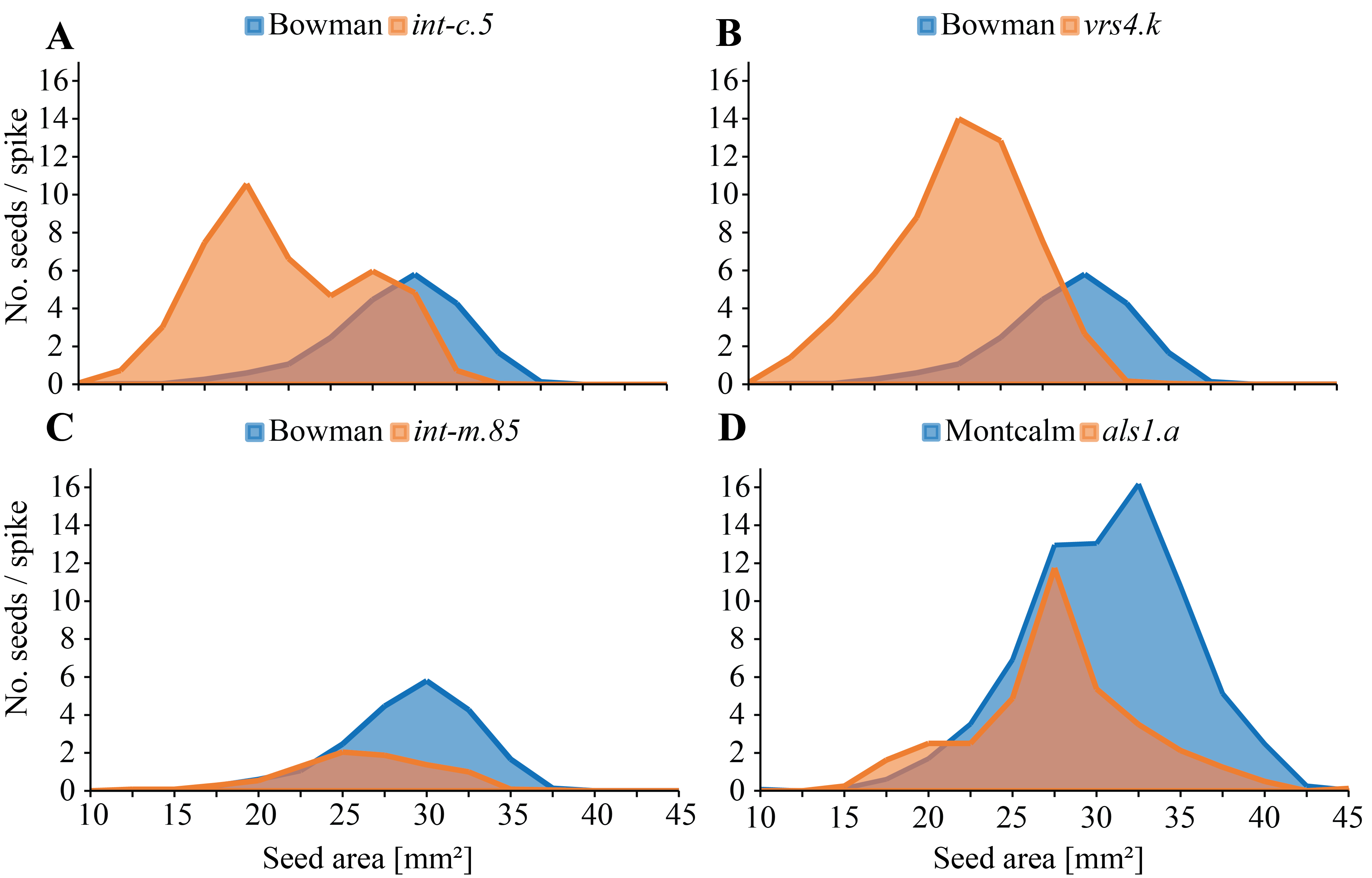

Supplement: S1 Fig — Graphs show the distribution of the seed area ranging between 10–45 mm2, n ≥ 15 spikes. (A) int-c.5, (B) vrs4.k, and (C) int-m.85 in Bowman background. (D) als1.a compared to Montcalm. All parameters were derived from plants grown outdoors. (TIF) [file pone.0140246.s001.tif]

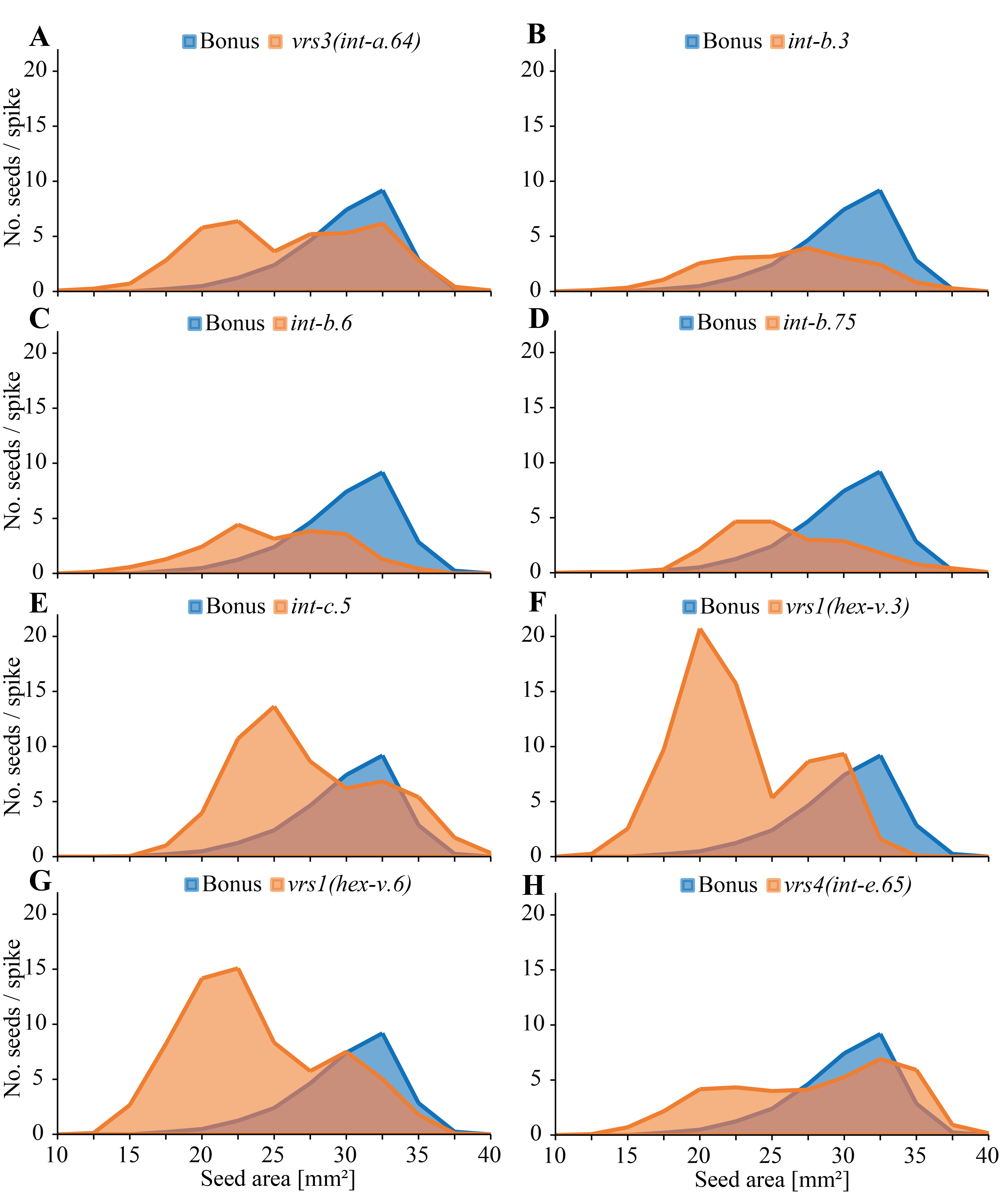

Supplement: S2 Fig — Graphs show distribution of the seed area ranging between 10–45 mm2, n ≥ 8 spikes. (A) vrs3(int-a.64), (B) int-b.3, (C), int-b.6, (D) int-b.75, (E) int-c.5, (F) vrs1(hex-v.3), (G) vrs1(hex-v.6), and (H) vrs4(int-e.65). All parameters were derived from plants grown outdoors. (TIF) [file pone.0140246.s002.tif]

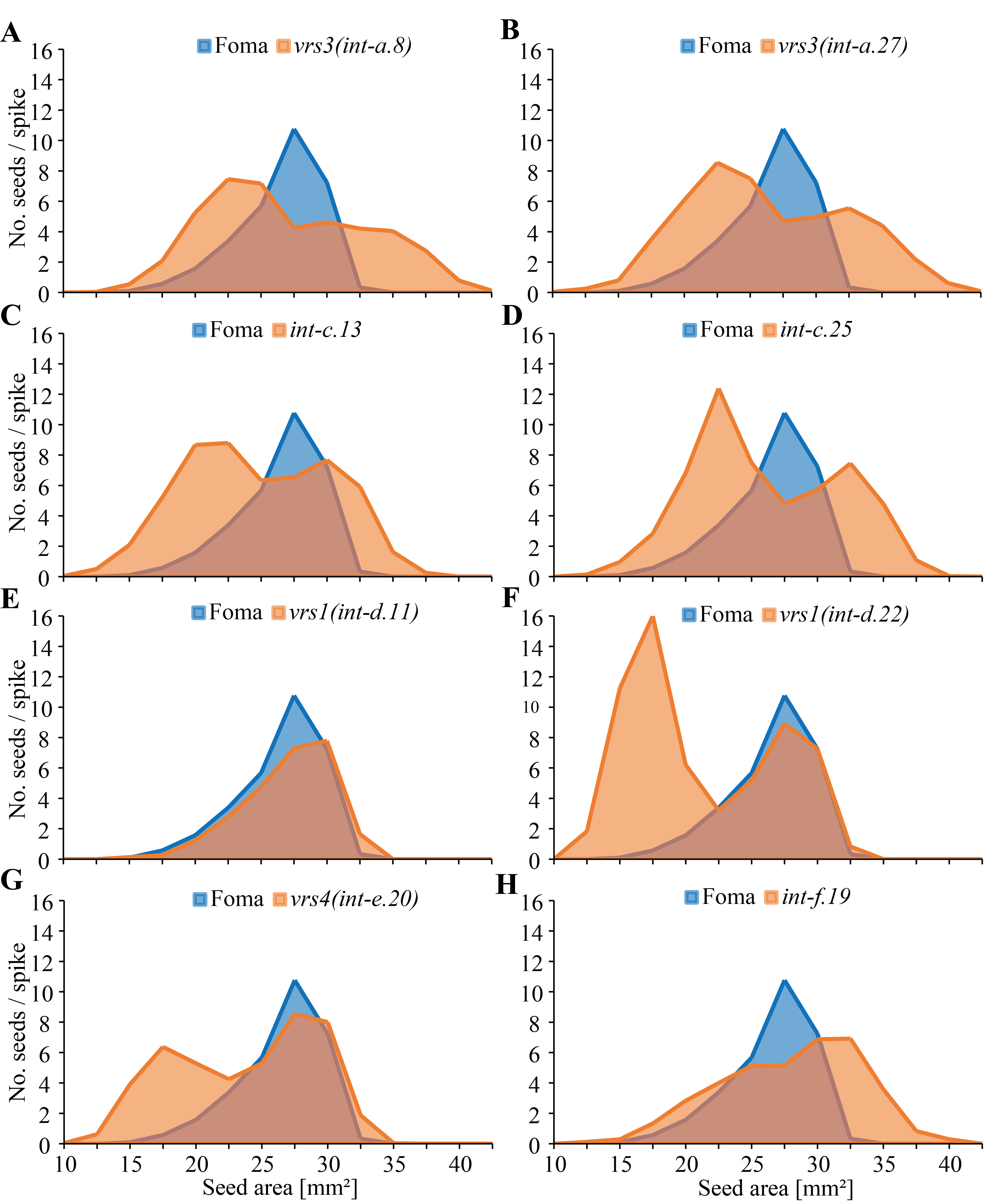

Supplement: S3 Fig — Graphs show the distribution of the seed area ranging between 10–45 mm2, n ≥ 8 spikes. (A) vrs3(int-a.8), (B) vrs3(int-a.27), (C) int-c.13, (D) int-c.25, (E) vrs1(int-d.11), (F) vrs1(int-d.22), (G) vrs4(int-e.20), and (H) int-f.19. All parameters were derived from plants grown outdoors. (TIF) [file pone.0140246.s003.tif]

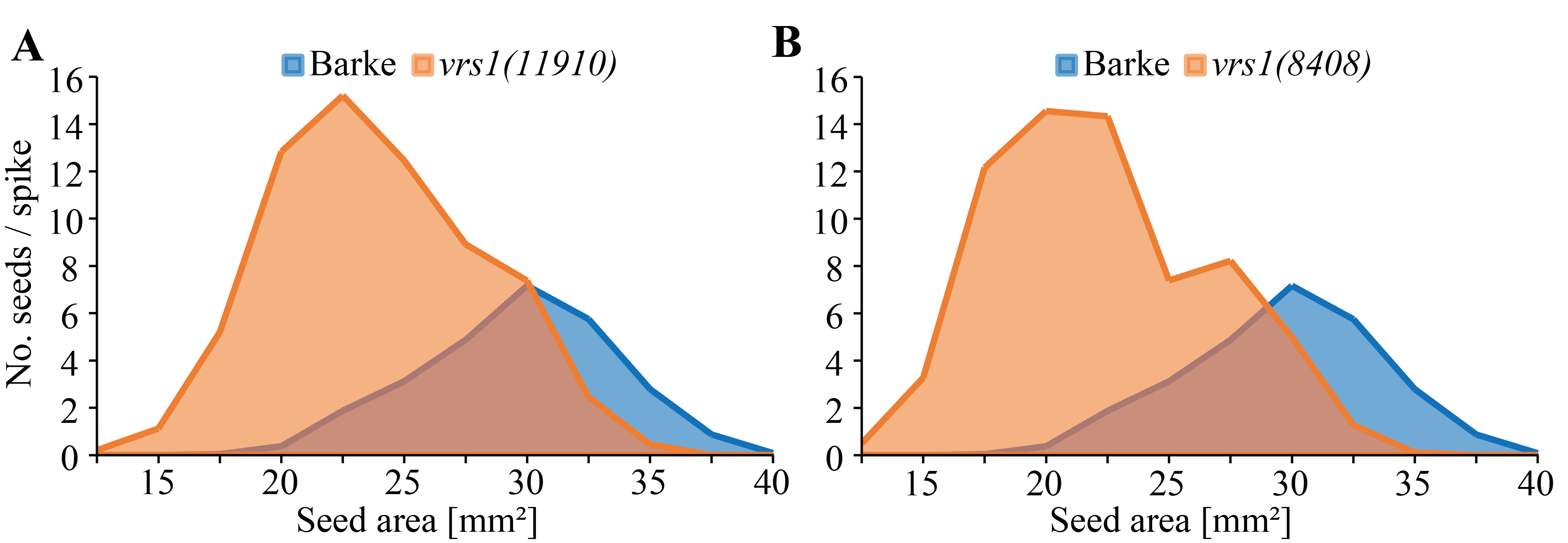

Supplement: S4 Fig — Graphs show the distribution of the seed area ranging between 10–45 mm2, n ≥ 8 spikes. (A) vrs1(11910) BC3S3, and (B) vrs1(8408) BC3S3. All parameters were derived from plants grown outdoors. (TIF) [file pone.0140246.s004.tif]

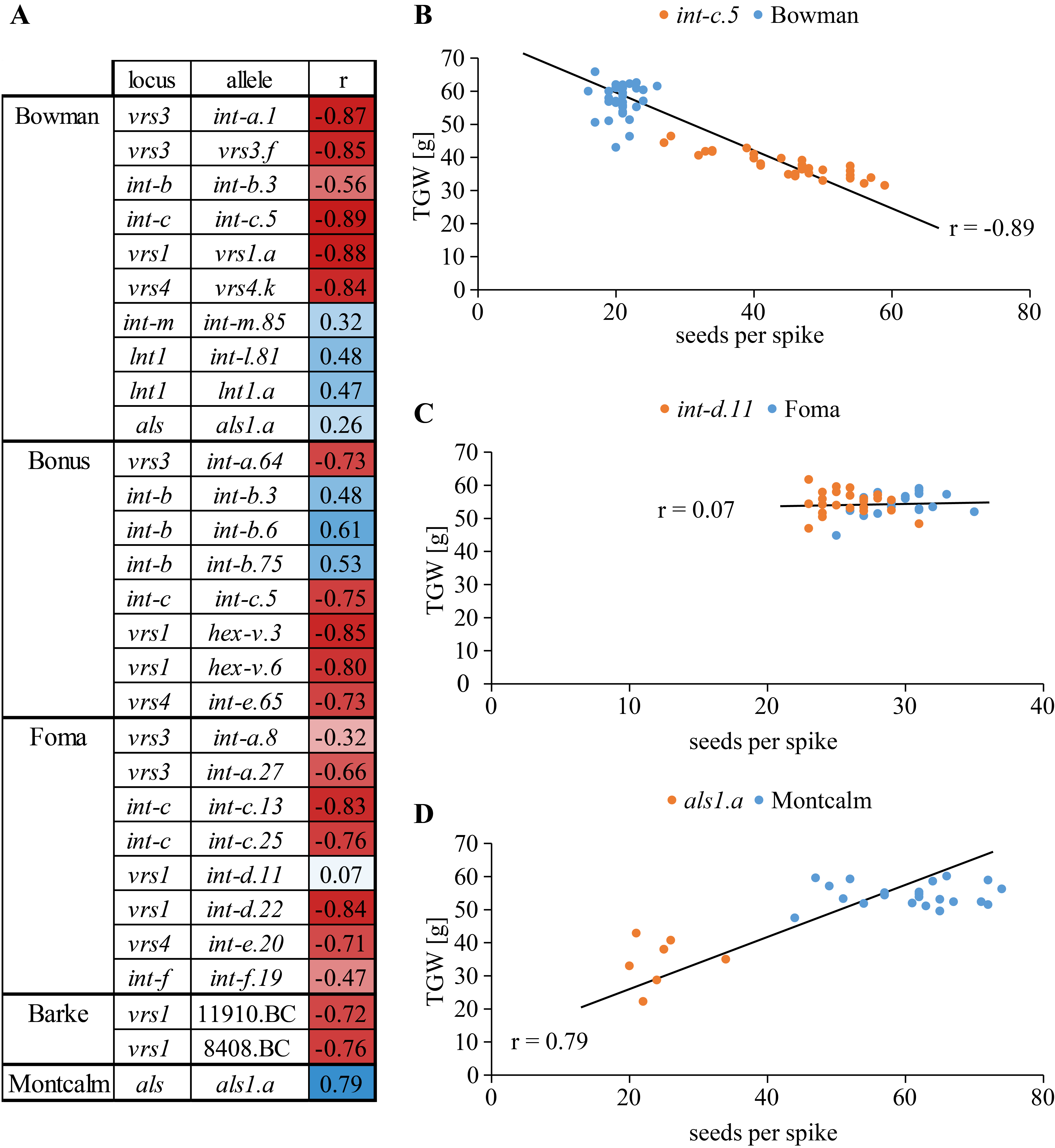

Supplement: S5 Fig — (A) Correlation coefficients (r) of seed number per spike vs. TGW between row type mutants and their respective wild types. (B-D) Exemplary x-y scatterplots depicting data points from mutant and wild type and their respective correlation coefficients. All parameters were derived from plants grown outdoors. (TIF) [file pone.0140246.s005.tif]

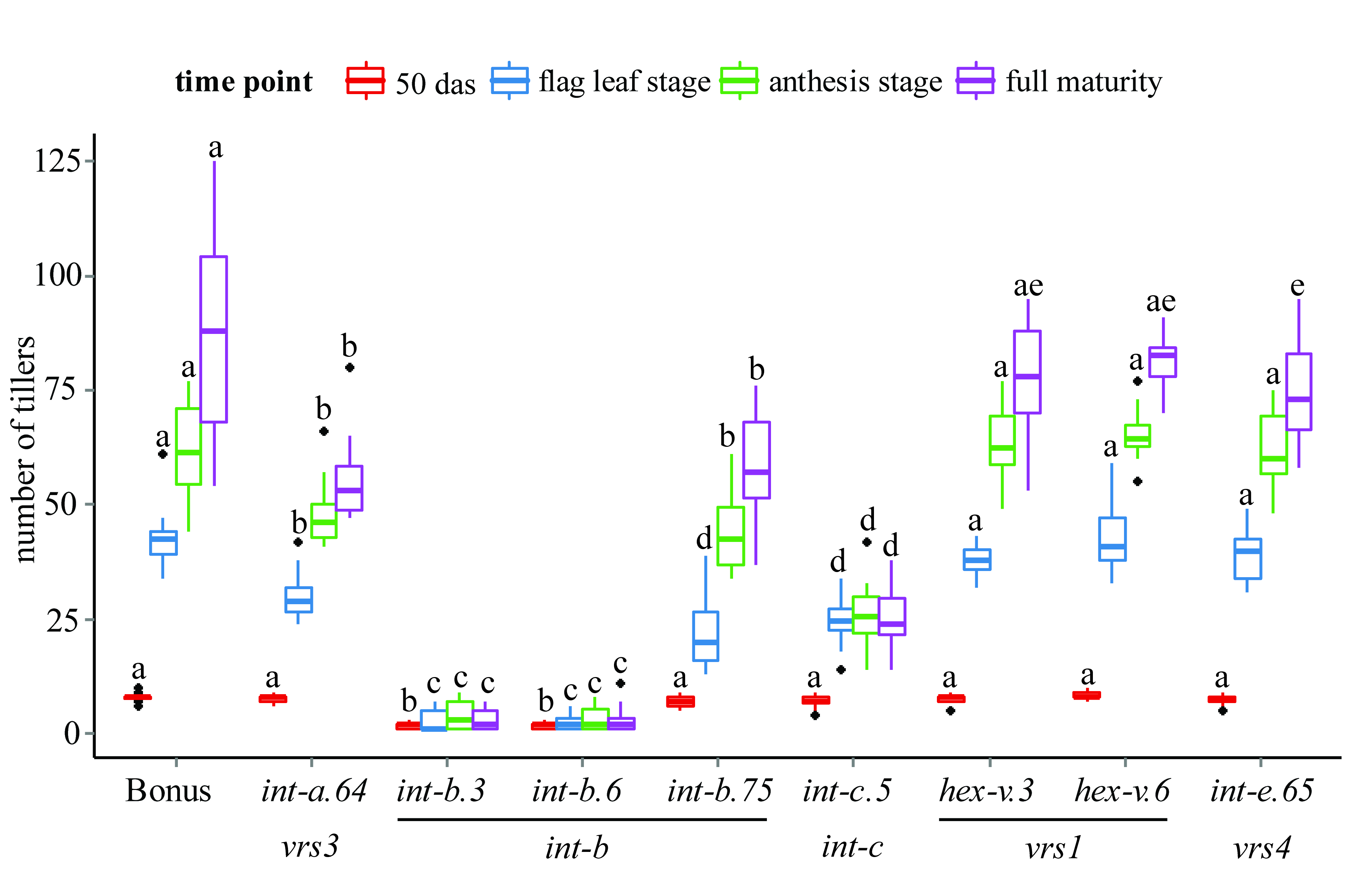

Supplement: S6 Fig — Letters indicate groups of genotypes with significant differences from one another at the same time point determined by a one-way ANOVA (p ≤ 0.05). Tiller numbers shown in this graph are derived from plants grown outdoors. (TIF) [file pone.0140246.s006.tif]

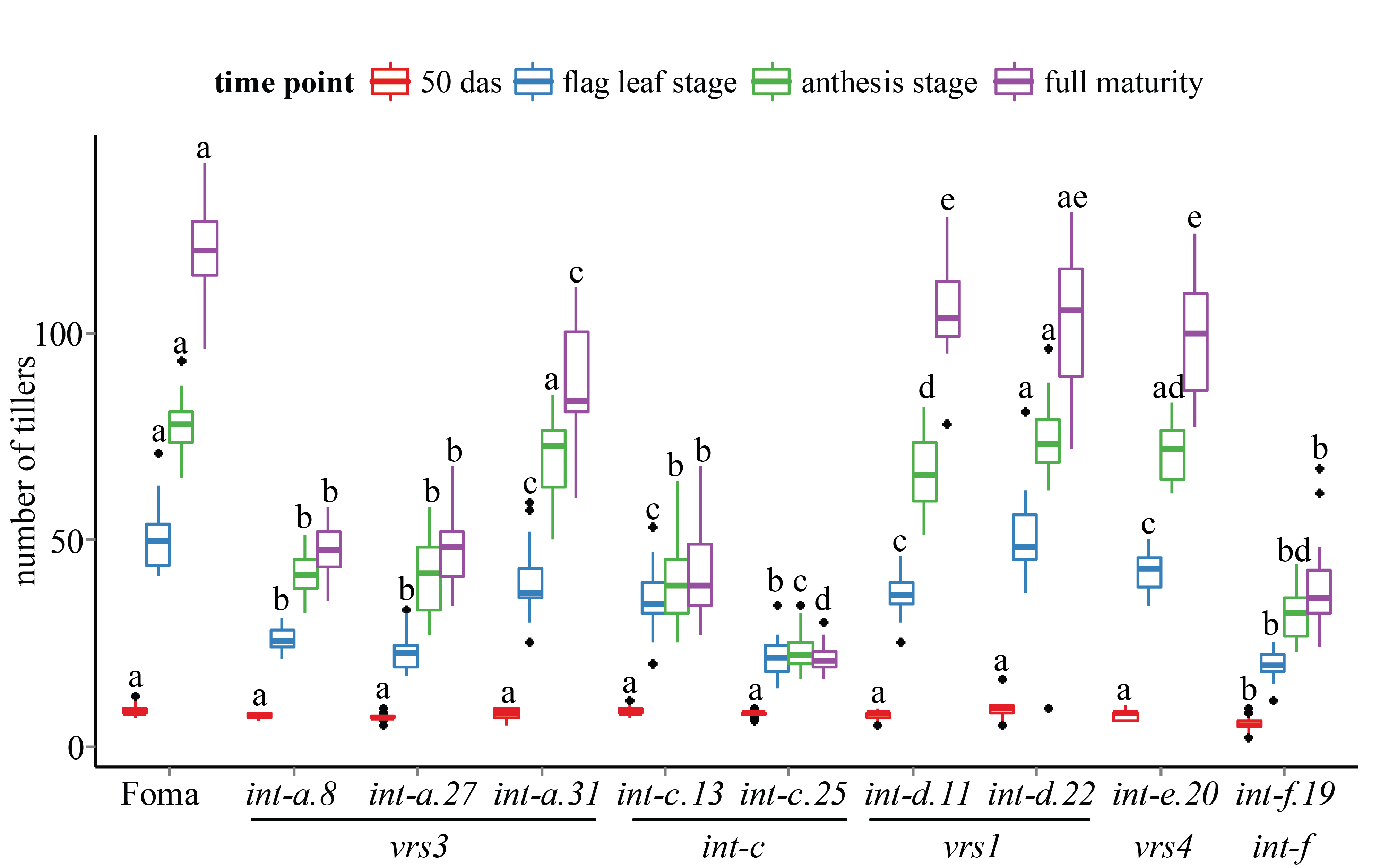

Supplement: S7 Fig — Letters indicate groups of genotypes with significant differences from one another at the same time point determined by a one-way ANOVA (p ≤ 0.05). Tiller numbers shown in this graph are derived from plants grown outdoors. (TIF) [file pone.0140246.s007.tif]

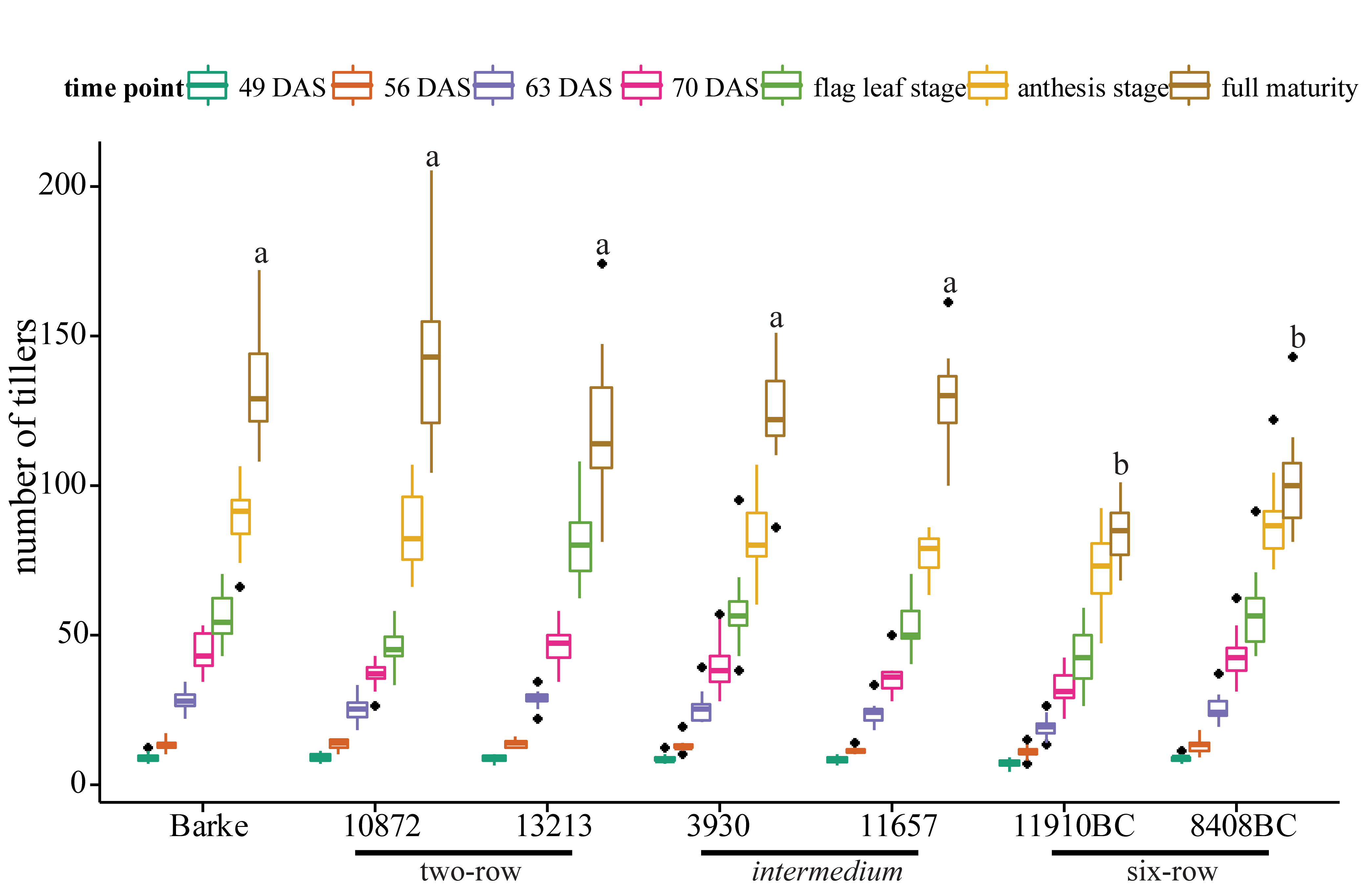

Supplement: S8 Fig — Letters indicate groups of genotypes with significant differences from one another at full maturity determined by a one-way ANOVA (p ≤ 0.05). Tiller numbers shown in this graph are derived from plants grown outdoors. (TIF) [file pone.0140246.s008.tif]

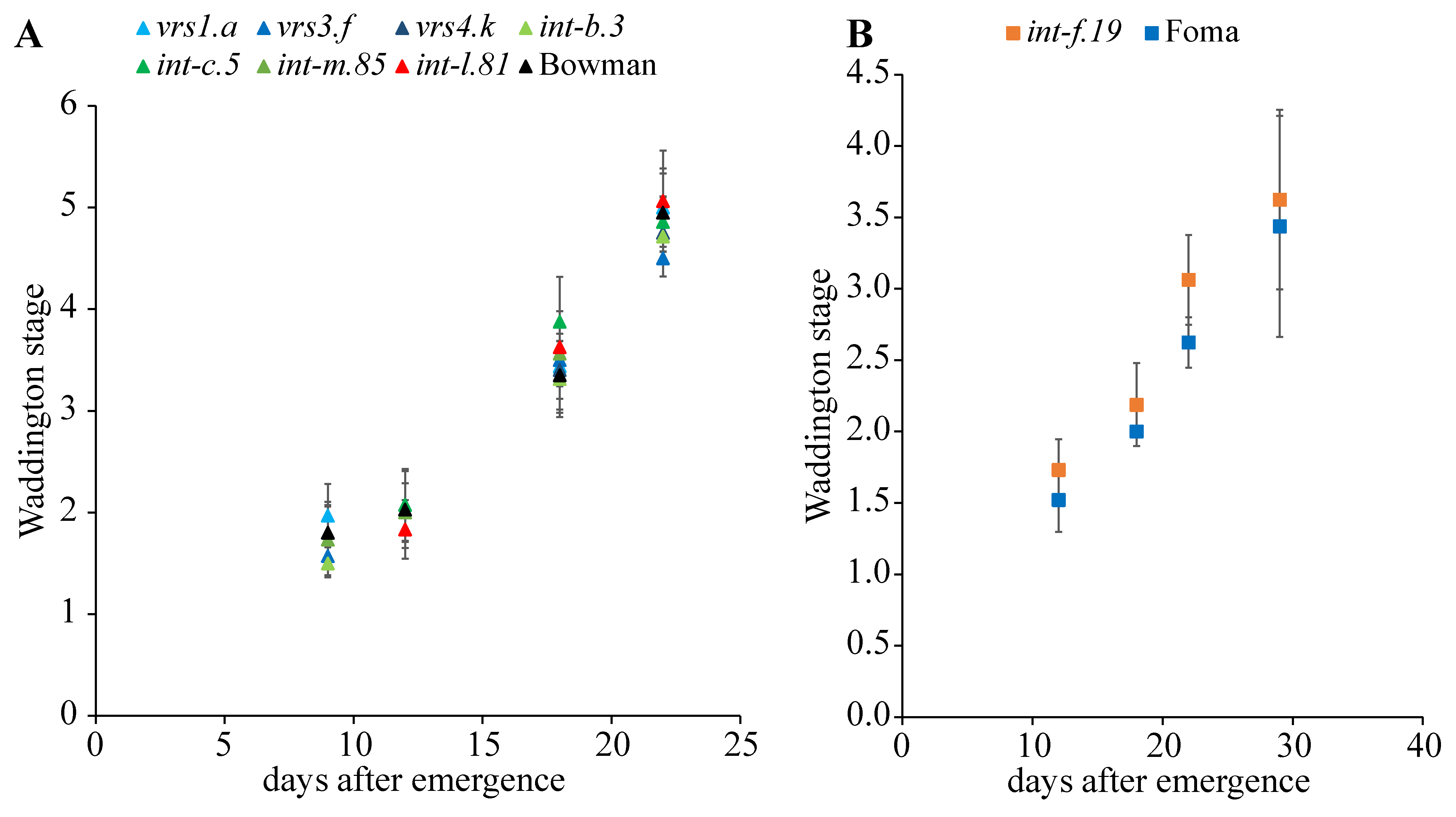

Supplement: S9 Fig — (A) vrs3, int-b, int-c, vrs1, vrs4, and lnt1 in Bowman backcross; and (B) int-f in Foma background. Development was staged according to Waddington et al. [27]. Error bars represent standard deviation. (TIF) [file pone.0140246.s009.tif]

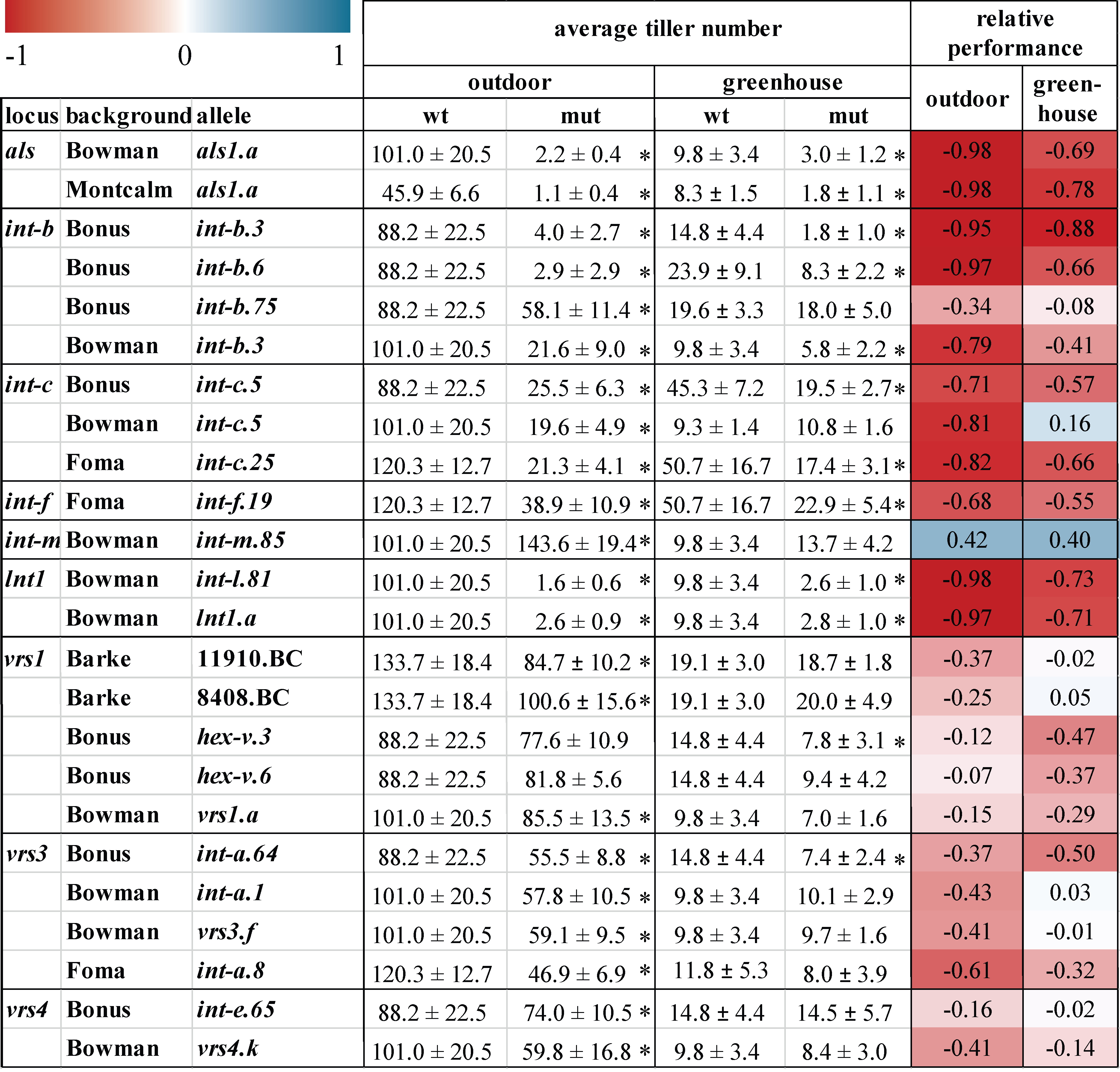

Supplement: S10 Fig — Relative performance was calculated using the respective wild type growing under the same conditions. In case multiple experiments were performed under greenhouse conditions, only results from one representative experiment are shown. Results from all individual experiments can be found in S1 File. Stars (*) indicate a significant difference between the mutant and wild type determined by a one-way ANOVA (p ≤ 0.05). (TIF) [file pone.0140246.s010.tif]

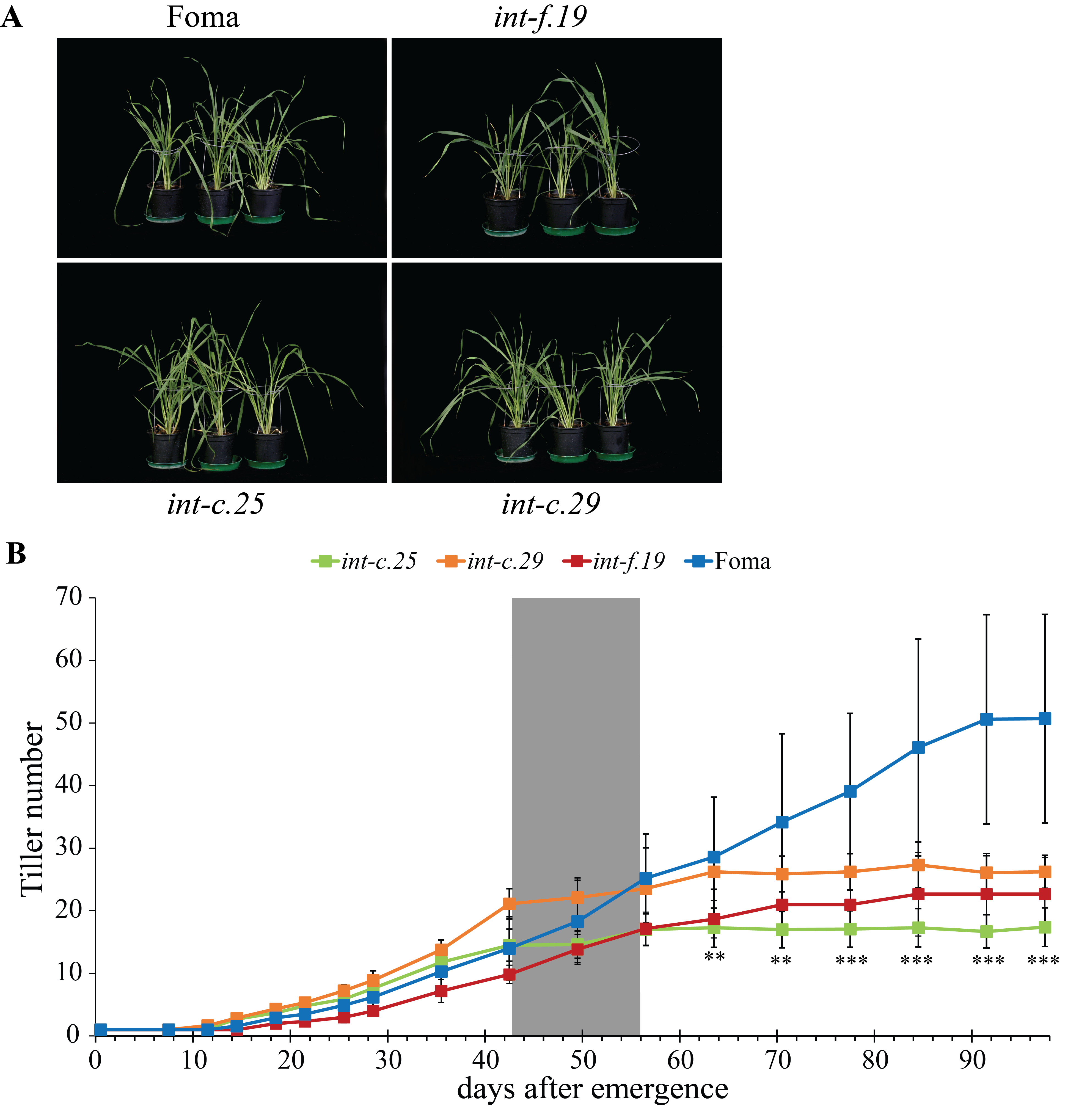

Supplement: S11 Fig — Ten biological replicates per genotype were grown in a controlled greenhouse under long day (LD) conditions. (A) Pictures of three representative plants per genotype were taken at 32 DAE. (B) This plot shows the tillering behavior of int-c and -f mutants from emergence until the age of 13 weeks after emergence. The gray area indicates the time period when plants reached flag leaf stage. The different genotypes within the same background did not differ significantly in flowering time. Bars indicate ± standard deviation. *Indicate significant difference (p≤ 0.05) when compared to Bowman background in int-c.29 and int-f.19 (**) or in int-c.25, int-c.29 and int-f.19 (***). (TIF) [file pone.0140246.s011.tif]

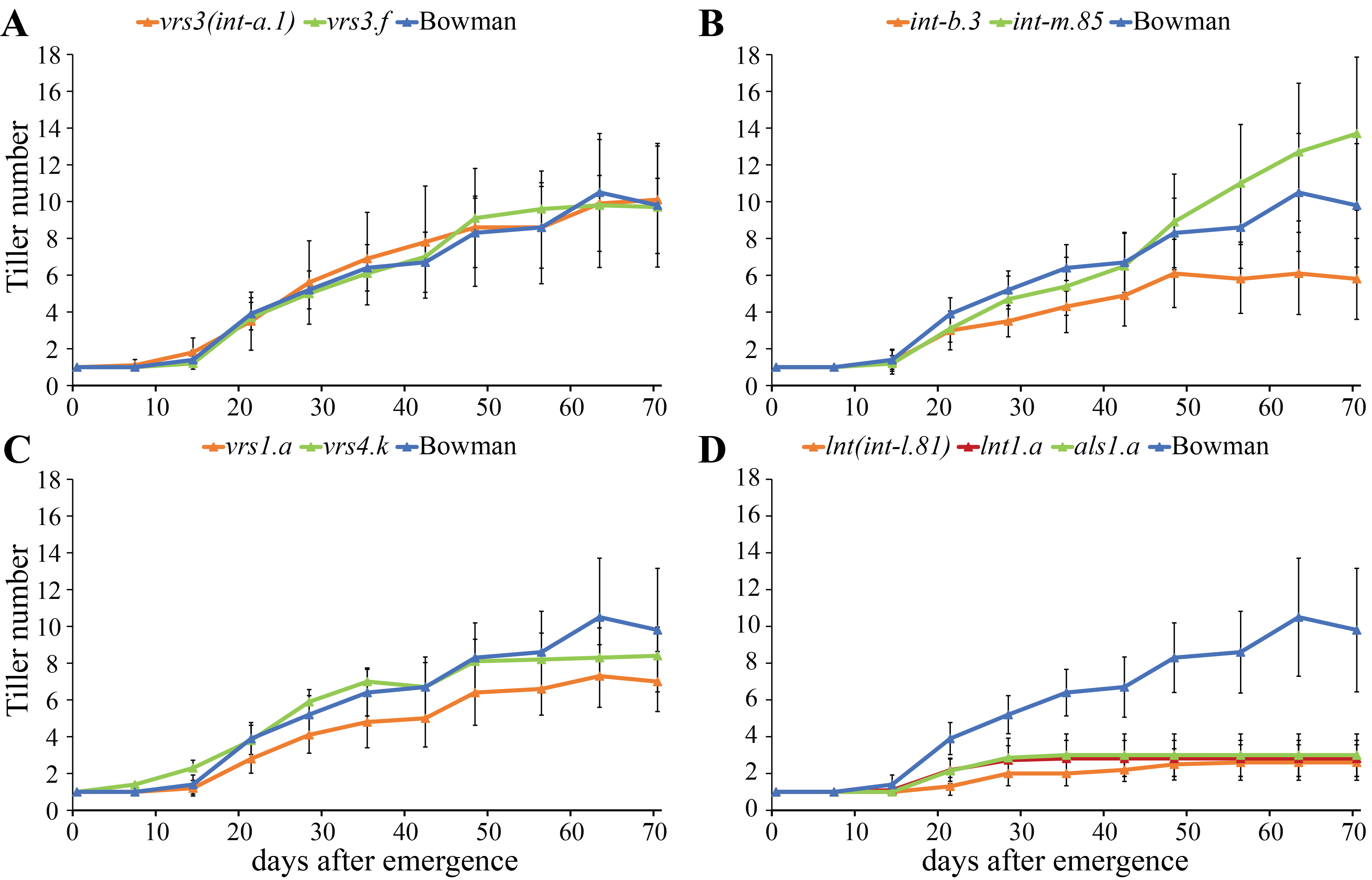

Supplement: S12 Fig — Ten biological replicates per genotype were grown in a controlled greenhouse under long day (LD) conditions. These plots show the tillering behavior of different row type mutants in Bowman background compared to Bowman until the age of ten weeks after emergence. (A) vrs3(int-a.1) and vrs3.f; (B) int-b.3 and int-m.85; (C) vrs1.a and vrs4.k; and (D) lnt1(int-l.81), lnt1.a and als1.a. Bars indicate ± standard deviation. Statistical differences are shown in S5 Table. (TIF) [file pone.0140246.s012.tif]
