# Supplementary material for: Mutations in Barley Row Type Genes Have Pleiotropic Effects on Shoot Branching
Source: PLoS One. 2015 Oct 14;10(10):e0140246. doi: 10.1371/journal.pone.0140246 (PMC4605766; doi:10.1371/journal.pone.0140246)
Supplement: S1 Table — (DOCX) [file pone.0140246.s015.docx]

**Table S 1: Names and alleles of *intermedium* and *vrs* genes used in this study.**

| **synonymous gene names** | **allele name** | **background** | **seed stock number** | **obtained from^1^** | **mutation** | **effect** | **published^2^** | **row type** | **used in experiment** | |
| --- | --- | --- | --- | --- | --- | --- | --- | --- | --- | --- |
|  |  |  |  |  |  |  |  |  | **field** | **greenhouse** |
| *vrs1, int-d, hex-v, HvHox1* | *vrs1.a* | Bowman (backcross) | GSHO 1907 | USDA | C 1020 G | F 075 L | [1] | six | **√** | **√** |
|  | *int-d.11* | Foma | NGB 115429 | NordGen | T 1476 A | C 194 S | [1] | *intermedium* | **√** |  |
|  | *int-d.22* | Foma | NGB 115440 | NordGen | G 1124 A | R 110 H | [1] | *intermedium* | **√** |  |
|  | *hex-v.3* | Bonus | NGB 115545 | NordGen | deletion | deletion | [1] | six | **√** | **√** |
|  | *hex-v.6* | Bonus | NGB 115547 | NordGen | n.d. | n.d. |  | six | **√** | **√** |
|  | 11910 | Barke BC_3_S_3_ | 11910-1 | S. Gottwald | G 0856 A | splice junction | [2] | six | **√** | **√** |
|  | 10872 | Barke M_4_ | 10872-1 | N. Stein | C 0968 T | P 058 L | [2] | two | **√** |  |
|  | 3930 | Barke M_4_ | 3930-1 | N. Stein | G 1039 A | E 082 K | [2] | *intermedium* | **√** |  |
|  | 8408 | Barke BC_3_S_3_ | 8408-1 | S. Gottwald | T 1079 A | L 095 Q | [2] | six | **√** | **√** |
|  | 11657 | Barke M_4_ | 11657-1 | N. Stein | G 1115 A | R 107 H | [2] | *intermedium* | **√** |  |
|  | 13213 | Barke M_4_ | 13213-1 | N. Stein | G 1471 A | G 192 E | [2] | two | **√** |  |
| *vrs3, int-a* | *vrs3.f* | Bowman (backcross) | GSHO 2056 | USDA | n.d. | n.d. |  | six | **√** | **√** |
|  | *int-a.1* | Bowman (backcross) | GSHO 2055 | USDA | n.d. | n.d. |  | six | **√** | **√** |
|  | *int-a.8* | Foma | NGB 115426 | NordGen | n.d. | n.d. |  | *intermedium* | **√** | **√** |
|  | *int-a.27* | Foma | NGB 115445 | NordGen | n.d. | n.d. |  | *intermedium* | **√** |  |
|  | *int-a.31* | Foma | NGB 115449 | NordGen | n.d. | n.d. |  | *intermedium* | **√** |  |
|  | *int-a.64* | Bonus | NGB 115482 | NordGen | n.d. | n.d. |  | *intermedium* | **√** | **√** |
| *vrs4, int-e, HvRA2* | *vrs4.k* | Bowman (backcross) | GSHO 1986 | USDA | C 072 del | nonsense | [3] | six | **√** | **√** |
|  | *int-e.20* | Foma | NGB 115438 | NordGen | n.d. | n.d. | [3] | *intermedium* | **√** |  |
|  | *int-e.65* | Bonus | NGB 115483 | NordGen | G 068 A | G 023 E | [3] | *intermedium* | **√** | **√** |
| *int-c, HvTB1* | *int-c.5* | Bowman (backcross) | GSHO 2003 | USDA | C 882 del | frame shift | [4] | *intermedium* | **√** | **√** |
|  | *int-c.5* | Bonus | NGB 115423 | NordGen | C 882 del | frame shift | [4] | *intermedium* | **√** | **√** |
|  | *int-c.13* | Foma | NGB 115431 | NordGen | A 443 T | Q 148 L | [4] | *intermedium* | **√** |  |
|  | *int-c.25* | Foma | NGB 115443 | NordGen | A 298 T | K 100 STOP | [4] | *intermedium* | **√** | **√** |
|  | *int-c.29* | Foma | NGB 115447 | NordGen | n.d. | n.d. | [4] | *intermedium* |  | **√** |
| *int-b* | *int-b.3* | Bowman (backcross) | GSHO 2129 | USDA | n.d. | n.d. |  | *intermedium* | **√** | **√** |
|  | *int-b.3* | Bonus | NGB 115421 | NordGen | n.d. | n.d. |  | *intermedium* | **√** | **√** |
|  | *int-b.6* | Bonus | NGB 115424 | NordGen | n.d. | n.d. |  | *intermedium* | **√** | **√** |
|  | *int-b.75* | Bonus | NGB 115493 | NordGen | n.d. | n.d. |  | *intermedium* | **√** | **√** |
| *int-f* | *int-f.19* | Bowman (backcross) | GSHO 2270 | USDA | n.d. | n.d. |  | *intermedium* |  | **√** |
|  | *int-f.19* | Foma | NGB 115437 | NordGen | n.d. | n.d. |  | *intermedium* | **√** | **√** |
| *int-m* | *int-m.85* | Bowman (backcross) | GSHO 2273 | USDA | n.d. | n.d. |  | *intermedium* | **√** | **√** |
| *int-l, lnt1* | *int-l.81* | Bowman (backcross) | GSHO 1961 | USDA | deletion | deletion | [5] | two | **√** | **√** |
|  | *lnt1.a* | Bowman (backcross) | GSHO 1984 | USDA | C 1199 del | frame shift | [5] | two | **√** | **√** |
| *als* | *als1.a* | Montcalm | GSHO 1065 | USDA | n.d. | n.d. |  | six | **√** | **√** |
|  | *als1.a* | Bowman (backcross) | GSHO 1990 | USDA | n.d. | n.d. |  | two | **√** | **√** |
| parent |  | Barke |  |  |  |  |  | two | **√** | **√** |
|  |  | Bonus | GSHO 464 |  |  |  |  | two | **√** | **√** |
|  |  | Bowman | PI 483237 |  |  |  |  | two | **√** | **√** |
|  |  | Foma | CIho 11333 |  |  |  |  | two | **√** | **√** |
|  |  | Montcalm | CIho 7149 |  |  |  |  | six | **√** | **√** |

Allele names and backgrounds of each line used. Bowman (backcross): original mutation was in a different background and was introduced into cv. Bowman by multiple backcrosses [6]. *vrs1* TILLING lines in cv. Barke have been described previously: [2]. n.d. = not determined since the underlying gene is not known.

^1^: USDA – National Small Grains Collection, U.S. Department of Agriculture; NordGen – seed originally obtained from the Nordic Genetic Resource Centre (NordGen); S. Gottwald – Department of Plant Breeding, Justus Liebig University Gießen, Germany; N. Stein – Genebank Department, Leibnitz-Institut für Pflanzengenetik und Kulturpflanzenforschung (IPK) Gatersleben, Germany. ^2^: Mutation originally published in.

1. Komatsuda T, Pourkheirandish M, He CF, Azhaguvel P, Kanamori H, et al. (2007) Six-rowed barley originated from a mutation in a homeodomain-leucine zipper I-class homeobox gene. Proceedings of the National Academy of Sciences of the United States of America 104: 1424-1429.

2. Gottwald S, Bauer P, Komatsuda T, Lundqvist U, Stein N (2009) TILLING in the two-rowed barley cultivar 'Barke' reveals preferred sites of functional diversity in the gene HvHox1. BMC research notes 2: 258-258.

3. Koppolu R, Anwar N, Sakuma S, Tagiri A, Lundqvist U, et al. (2013) Six-rowed spike4 (Vrs4) controls spikelet determinacy and row-type in barley. Proceedings of the National Academy of Sciences of the United States of America 110: 13198-13203.

4. Ramsay L, Comadran J, Druka A, Marshall DF, Thomas WTB, et al. (2011) INTERMEDIUM-C, a modifier of lateral spikelet fertility in barley, is an ortholog of the maize domestication gene TEOSINTE BRANCHED 1. Nature Genetics 43: 169-U125.

5. Dabbert T, Okagaki RJ, Cho S, Heinen S, Boddu J, et al. (2010) The genetics of barley low-tillering mutants: low number of tillers-1 (lnt1). Theoretical and Applied Genetics 121: 705-715.

6. Druka A, Franckowiak J, Lundqvist U, Bonar N, Alexander J, et al. (2011) Genetic Dissection of Barley Morphology and Development. Plant Physiology 155: 617-627.
