# Supplementary material for: Mutations in Barley Row Type Genes Have Pleiotropic Effects on Shoot Branching
Source: PLoS One. 2015 Oct 14;10(10):e0140246. doi: 10.1371/journal.pone.0140246 (PMC4605766; doi:10.1371/journal.pone.0140246)
Supplement: S2 Table — (DOCX) [file pone.0140246.s016.docx]

Table S2: **Verification of the mutations in the *vrs1* gene.**

| **Name^1^** | **background** | **published mutation^2^** | **mutation found** | **effect** | **row type** |
| --- | --- | --- | --- | --- | --- |
| *hex-v.3* | Bonus | 1-2147 deletion | smaller deletion  (1-423) | Null | six |
| *hex-v.6* | Bonus | n.d. | A 0694 G,  T 0873 G,  G 1352 del,  C 1589 T | D 008 G,  D 026 E,  fame shift,  no effect (UTR) | six |

^1^ Only *vrs1* loci where the published mutations could not be verified are enlisted.

^2^ published in [1]

1. Komatsuda T, Pourkheirandish M, He CF, Azhaguvel P, Kanamori H, et al. (2007) Six-rowed barley originated from a mutation in a homeodomain-leucine zipper I-class homeobox gene. Proceedings of the National Academy of Sciences of the United States of America 104: 1424-1429.
