# Supplementary material for: Mutations in Barley Row Type Genes Have Pleiotropic Effects on Shoot Branching
Source: PLoS One. 2015 Oct 14;10(10):e0140246. doi: 10.1371/journal.pone.0140246 (PMC4605766; doi:10.1371/journal.pone.0140246)
Supplement: S5 Table — (DOCX) [file pone.0140246.s019.docx]

**Table S5: Genetic and environmental effect of row type mutants on tillering.**

|  | **source** | **DF** | **R2 (%)** | **F value** | **P value** |
| --- | --- | --- | --- | --- | --- |
| *vrs3* | gene | 1 | 15.3 | 320 | *** |
|  | gene (allele) | 7 | 2.9 | 12 | *** |
|  | environment | 1 | 62.8 | 1312 | *** |
|  | gene x environment | 1 | 4.8 | 100 | *** |
|  | gene(allele) x environment | 1 | 1.7 | 7 | *** |
| *int-b* | gene | 1 | 27.0 | 410 | *** |
|  | gene (allele) | 3 | 8.6 | 43 | *** |
|  | environment | 1 | 3.9 | 499 | *** |
|  | gene x environment | 1 | 1.8 | 269 | *** |
|  | gene(allele) x environment | 1 | 10.0 | 25 | *** |
| *int-c* | gene | 1 | 30.1 | 531 | *** |
|  | gene (allele) | 2 | 5.3 | 31 | *** |
|  | environment | 1 | 23.2 | 409 | *** |
|  | gene x environment | 1 | 14.9 | 264 | *** |
|  | gene(allele) x environment | 2 | 1.9 | 12 | *** |
| *vrs1* | gene | 1 | 1.6 | 48 | *** |
|  | gene (allele) | 6 | 0.3 | 14 | *** |
|  | environment | 1 | 78.1 | 2328 | *** |
|  | gene x environment | 1 | 1.1 | 34 | *** |
|  | gene(allele) x environment | 1 | 1.7 | 9 | *** |
| *vrs4* | gene | 1 | 4.3 | 52 | *** |
|  | gene (allele) | 4 | 0.6 | 4 | * |
|  | environment | 1 | 63.6 | 767 | *** |
|  | gene x environment | 1 | 1.6 | 19 | *** |
|  | gene(allele) x environment | 2 | 2.1 | 13 | *** |

|  | **source** | **DF** | **R2 (%)** | **F value** | **P value** |
| --- | --- | --- | --- | --- | --- |
| *int-f* | gene | 1 | 39.4 | 202 | *** |
|  | environment | 1 | 24.3 | 125 | *** |
|  | gene x environment | 1 | 9.3 | 48 | *** |
| *int-m* | gene | 1 | 3.2 | 55 | *** |
|  | environment | 1 | 87.4 | 1476 | *** |
|  | gene x environment | 1 | 3.2 | 54 | *** |
| *lnt1* | gene | 1 | 55.9 | 1458 | *** |
|  | gene (allele) | 2 | 0.0 | 0 | n.s. |
|  | environment | 1 | 30.1 | 786 | *** |
|  | gene x environment | 1 | 40.0 | 1042 | *** |
|  | gene(allele) x environment | 1 | 0.0 | 0 | n.s. |
| *als* | gene | 1 | 34.6 | 218 | *** |
|  | environment | 1 | 21.2 | 133 | *** |
|  | gene x environment | 1 | 22.8 | 143 | *** |

**Table S5:** Continued
