# Supplementary material for: Mutations in Barley Row Type Genes Have Pleiotropic Effects on Shoot Branching
Source: PLoS One. 2015 Oct 14;10(10):e0140246. doi: 10.1371/journal.pone.0140246 (PMC4605766; doi:10.1371/journal.pone.0140246)
Supplement: S6 Table — (DOCX) [file pone.0140246.s020.docx]

**Table S6:** ***int-c* mutants do not change their tiller number significantly after flag leaf stage.**

| **Background** | **mutation** | **tiller number at^1^** | | **Significance^2^** |
| --- | --- | --- | --- | --- |
|  |  | **flag leaf stage** | **maturity** |  |
| **Bowman** | **Bowman** | 6.5 ± 1.3 | 9.3 ± 1.4 | *** |
|  | ***int-c.5*** | 9.9 ± 1.1 | 10.8 ± 1.6 | n.s. |
|  | ***int-f.19*** | 5.5 ± 0.8 | 7.6 ± 0.8 | ** |
| **Bonus** | **Bonus** | 19.8 ± 4.0 | 45.3 ± 7.0 | *** |
|  | ***int-c.5*** | 18.1 ± 2.7 | 19.1 ± 2.4 | n.s. |
| **Foma** | **Foma** | 18.3 ± 6.6 | 50.6 ± 16.7 | *** |
|  | ***int-c.25*** | 14.6 ± 2.2 | 16.7 ± 2.7 | n.s. |
|  | ***int-c.29*** | 22.1 ± 3.2 | 26.1 ± 2.7 | n.s. |
|  | ***int-f.19*** | 13.8 ± 2.4 | 22.9 ± 6.0 | n.s. |

^1^Average values ± standard deviation are shown, n = 10 plants

^2^Significance levels of differences between the mutant and their respective parental cultivar were calculated using a student’s t-test (*: *p* ≤ 0.05, **: *p* ≤ 0.01, ***: *p* ≤ 0.001, n.s.: not significant).
